# Cu(I)-catalyzed *N*,*N*’-diarylation of natural diamines and polyamines with aryl iodides

**DOI:** 10.3762/bjoc.11.250

**Published:** 2015-11-24

**Authors:** Svetlana Petrovna Panchenko, Alexei Dmitrievich Averin, Maksim Viktorovich Anokhin, Olga Aleksandrovna Maloshitskaya, Irina Petrovna Beletskaya

**Affiliations:** 1 Department of Chemistry, Lomonosov Moscow State University, Leninskie Gory 1-3, Moscow, 119991, Russia

**Keywords:** amination, aryl amines, aryl iodides, copper catalysis, polyamines

## Abstract

The Cu(I)-catalyzed *N*,*N*’-diarylation of natural diamines and polyamines such as putrescine, cadaverine, spermine, spermidine and their homologues is described. Aryl iodides bearing electron-donating and electron-withdrawing groups have been employed in the study. The CuI/2-(isobutyryl)cyclohexanone/DMF catalytic system has found to be more efficient in the diarylation of diamines and spermine while the CuI/L-proline/EtCN system proved to be preferable for the diarylation of other tri- and tetraamines like spermidine, norspermidine and norspermine.

## Introduction

Natural diamines and polyamines like putrescine (butane-1,4-diamine), spermidine (*N*^1^-(3-aminopropyl)butane-1,4-diamine) and spermine (*N*^1^,*N*^1'^-(butane-1,4-diyl)dipropane-1,3-diamine) are biologically active compounds which play crucial roles in the processes of cell proliferation, apoptosis and adaptation to stress impacts. The important biological processes occur also with the participation of such diamines as propane-1,3-diamine and cadaverine (pentane-1,5-diamine), and polyamines like norspermidine (*N*^1^-(3-aminopropyl)propane-1,3-diamine) and norspermine (*N*^1^,*N*^1'^-(propane-1,3-diyl)dipropane-1,3-diamine) [1]. In the early 1970s cancer cells were found to possess an excess of polyamines [2]. This fact initiated the studies of polyamines in the frames of molecular biology and biochemistry. It has been firmly established that *N*-derivatives are prospects for the creation of anticancer and antiviral medicaments. The majority of known di- and polyamine derivatives possess alkyl or benzyl substituents at the nitrogen atoms [3–8], however, the synthesis and investigation of *N*-aryl derivatives of polyamines have been addressed only recently [9–11]. For example, some *N,N’*-diphenyl-α,ω-diaminoalkanes have been found to possess respiratory stimulating effects [12], *N*-substituted putrescine and cadaverine have shown antiproliferative and cytotoxic activity [13–14], *N*-decyl and *N*-dodecyl derivatives of putrescine, *N*-(*p*-tolyl) derivatives of cadaverine and hexane-1,6-diamine have demonstrated affinity to NMDA receptors and antileishmanial activity [15–17].

Up to date no general non-catalytic approach to *N,N’*-diarylpolyamines has yet been described. Synthetic procedures are multistep [17–18] though sometimes they can be performed as one-pot syntheses [19]. Several catalytic approaches have been described in the literature. One of them employs an iridium-based catalyst with amidophosphonate as the ligand which allows to convert aminoalcohols into *N*-monoaryl-substituted diamines by the reaction with arylamines [20]. Another method uses a bimetallic catalyst (Pt–Sn/γ-Al_2_O_3_) in the reactions of diols with amines, and a valuable *N,N’*-diphenylhexane-1,6-diamine was obtained using this catalyst [21]. More traditional and convenient Pd(0)-catalyzed amination, proposed by Buchwald and Hartwig [22–23], was successfully applied in the synthesis of mono- and diaryl-substituted diamines and polyamines in the group of Beletskaya [24–27]. It has been shown that the secondary dialkylamino groups in linear polyamines are practically inert and this allows a selective arylation of terminal primary amino groups. The exchange of expensive palladium accompanied with toxic ligands for a much cheaper copper catalyst is one of the main trends in modern catalytic chemistry. However, in spite of numerous works dealing with Cu(I)-catalyzed arylation of monoamines, there are scarce examples of the synthesis of *N*-arylpolyamines using this method. Han and coworkers for example showed the possibility to synthesize *N*-aryldiamines using aryl iodides in the presence of CuCl under neat conditions [28], *N,N’*-diarylation of the simplest propane-1,3-diamine and butane-1,4-diamine was carried out using a CuI-metformin catalyst [29], monoarylation of mono- and diamines was studied using Cu_2_O and CuO nanoparticles and CuO microparticles [30].

We initiated our studies in this field by the elaboration of Cu(I)-catalyzed *N*-arylations and *N*,*N*’-diarylations of the model triamine, tetraamine and oxadiamine with aryl iodides and bromides [31] and elucidated some regularities of the catalytic *N*-heteroarylation of polyamines [32]. However, it has been found that to obtain a good result, the reaction conditions (ligand, solvent, temperature) should be adjusted for a certain aryl halide/polyamine pair. In the present study we decided to undertake a thorough investigation of the Cu(I)-catalyzed *N*,*N*’-diarylation of natural diamines and polyamines using aryl iodides with electron-donating and electron-withdrawing substituents in order to identify conditions for the synthesis of a wide range of perspective derivatives of these di- and polyamines.

## Results and Discussion

### *N*,*N*’-Diarylation of diamines

**Figure 1:**
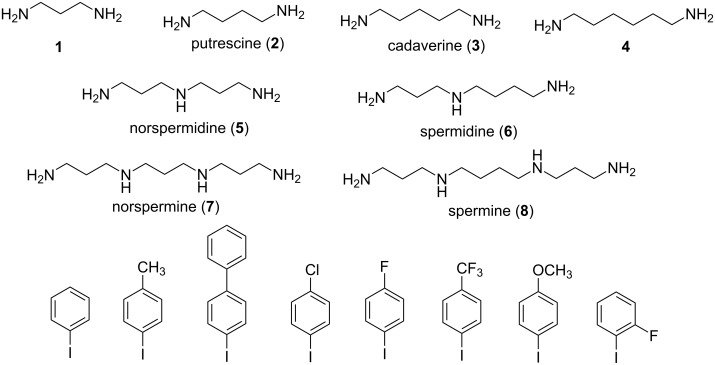
Diamines and polyamines studied in Cu(I)-catalyzed amination reactions.

The investigated diamines **1–4**, triamines **5**, **6** and tetraamines **7** and **8** as well as aryl iodides are presented on Figure 1. Di- and polyamines differ by the number of nitrogen atoms and methylene groups in the chain that dramatically influences their reactivity, as shown in our previous investigations. Aryl iodides differ by the electronic properties of the substituents and, in the case of 4- and 2-fluoroiodobenzene, by the steric hindrance at the reaction center. Also the choice of the substituents depends on the potential usefulness of corresponding *N,N’*-diaryl derivatives, for this purpose we referred to the data published in [30]. A special interest is paid to the compounds with fluorine and trifluoromethyl substituents as up to a quarter of pharmaceuticals contain fluorine in the aromatic or heteroaromatic ring. In this study we did not test substituted bromobenzenes as they were shown to be much less active than corresponding iodides in the copper-catalyzed amination of di- and polyamines providing mainly *N*-monoaryl derivatives [31].

**Scheme 1:**
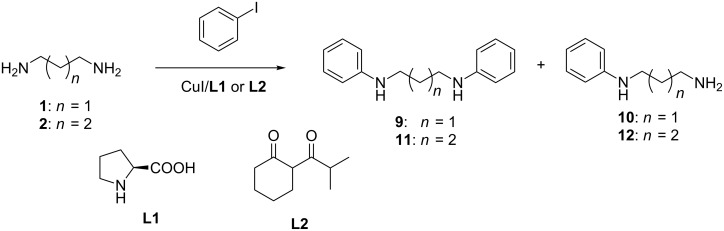
*N,N’*-Diarylation of the diamines **1** and **2**.

On the basis of our recent investigations, in order to obtain *N,N’*-diaryl derivatives, we employed the most suitable catalytic systems, CuI/L-proline (**L1**) and CuI/2-(isobutyryl)cyclohexanone (**L2**), EtCN or DMF were used as solvents and cesium carbonate (2.5 equiv) was taken as base (Scheme 1). The reactions were run under argon for ca. 24 h using 2.5 equiv of the aryl iodides with 0.5 M concentrations of polyamines. In the case of EtCN the reactions were refluxed (ca 100 °C), in the case of DMF they were run at 110 °C. Normal catalytic loading was 5 mol % CuI and 10 mol % ligand per 1 amino group.

At first we conducted the cross-coupling reactions between the simplest iodobenzene and diamines **1** and **2** to optimize the reaction conditions (Scheme 1, Table 1). As the resulting compounds **9**–**12** are described in the literature [29,33–35], it was possible to analyze the reaction mixtures by ^1^H and ^13^C NMR spectroscopy and in some cases the products were isolated by column chromatography on silica gel. For example, in the proton NMR spectrum of compound **9** two signals are observed in the aliphatic part: quintet at 1.94 ppm (2H) and triplet at 3.25 ppm (4H), while the spectrum of compound **10** is characterized by three signals: quintet at 1.76 ppm (2H), broad singlet at 2.86 ppm (2H) and triplet at 3.19 ppm (2H). The signals of two compounds do not overlap and can be easily integrated.

**Table 1:** Optimization of the reaction conditions for the *N*,*N*’-diarylation of diamines **1** and **2** with iodobenzene.

Entry	Amine	Catalytic system	CuI/L, mol %	*t*, °C	Products and yields, %^a^

1	**1**	CuI/**L1**/DMF	10/20	110	**9**, 40; **10**, 60
2	**1**	CuI/**L1**/EtCN	10/20	100	**9**, 45; **10**, 55
3	**1**	CuI/**L2**/DMF	10/20	110	**9**, 75; **10**, 25
4	**2**	CuI/**L1**/EtCN	10/20	100	**11**, 60 (43); **12**, 40
5	**2**	CuI/**L1**/Ph_3_P/EtCN	10/10/10	100	**11**, 30; **12**, 55
6	**2**	CuI/**L2**/Ph_3_P/DMF	10/10/10	110	**11**, 53 (41); **12**, 47

^a^Isolated yields are given in brackets.

When the reaction of diamine **1** with iodobenzene was carried out in the presence of the ligand **L1**, the yield of the target diphenyl derivative **9** did not exceed 40–45% (Table 1, entries 1 and 2), and mainly *N*-phenyl-substituted diamine **10** was formed. In the presence of the ligand **L2** the yield of **9** increased up to 75% (Table 1, entry 3). Diarylation of putrescine (**2**), on contrary, was more successful in the presence of the CuI/**L1**/EtCN catalytic system (Table 1, entry 4), and a similar result was obtained when using **L2** together with Ph_3_P (Table 1, entry 6), while employing **L1** with triphenylphosphine led to a low conversion into diphenyl derivative **11** (Table 1, entry 5). In general, diamine **2** was found to be less reactive than propane-1,3-diamine (**1**) in the coupling with iodobenzene.

**Scheme 2:**
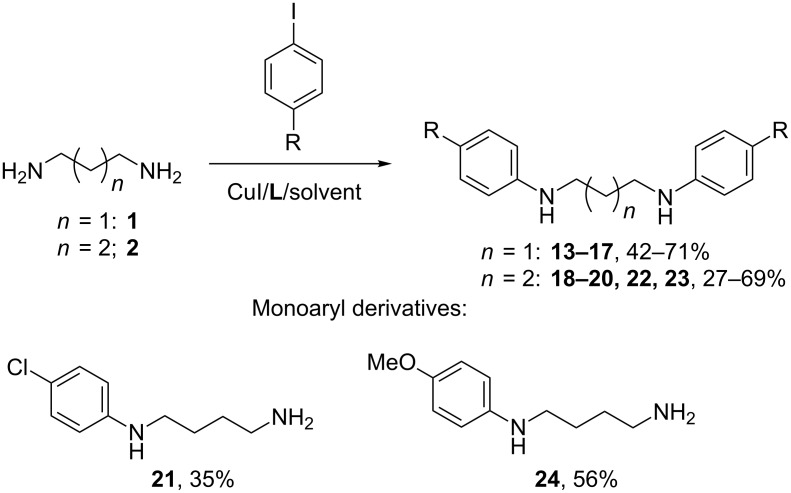
Arylation of the diamines **1** and **2**.

According to these preliminary results, we carried out all other reactions of diamines **1** and **2** with other aryl iodides using CuI/**L2**/DMF (10/20 mol %) catalytic system (Scheme 2, Table 2).

**Table 2:** Arylation of the diamines **1** and **2**.

Entry	Amine	R	Catalytic system	CuI/L, mol %	Products and yields, %^a^

1	**1**	Ph	CuI/**L2**/DMF	10/20	**13**, 56
2	**1**	Cl	CuI/**L2**/DMF	10/20	**14**, 71
3	**1**	F	CuI/**L2**/DMF	10/20	**15**, 61
4	**1**	CF_3_	CuI/**L2**/DMF	10/20	**16**, 42
5	**1**	OMe	CuI/**L2**/DMF	10/20	**17**, 56
6	**2**	Me	CuI/**L2**/Ph_3_P/DMF	10/10/10	**18**, 30^b^
7	**2**	Me	CuI/**L2**/DMF	20/40	**18**, 60
8	**2**	Ph	CuI/**L1**/EtCN	10/20	**19**, 46
9	**2**	Cl	CuI/**L2**/DMF	10/20	**20**, 14
10	**2**	Cl	CuI/**L2**/Ph_3_P/DMF	10/10/10	**20**, 27; **21**, 35
11	**2**	Cl	CuI/**L2**/Ph_3_P/DMF	20/20/20	**20**, 69
12	**2**	F	CuI/**L2**/Ph_3_P/DMF	20/20/20	**22**, 52
13	**2**	CF_3_	CuI/**L2**/Ph_3_P/DMF	10/10/10	**23**, 58
14	**2**	OMe	CuI/**L2**/Ph_3_P/DMF	10/10/10	**24**, 56

^a^Yields after chromatographic isolation. ^b^Yield in the reaction mixture.

The reactions with iodoarenes containing electron-withdrawing substituents ran successfully and corresponding diarylated products **13–16** were isolated in moderate to good yields (Table 2, entries 1–4). The difference in the preparative yields was due to the conditions of chromatographic isolation in each case. 4-Iodoanisole with a strong electron-donating substituent provided 56% yield of the diarylated compound **17** (Table 2, entry 5), while one could expect much lower reactivity of this compound compared to aryl iodides bearing acceptors. Diarylation of putrescine (**2**) was found to be more difficult (Scheme 2, Table 2). In the reactions with all aryl iodides we observed the formation of di- and monoarylated products in the reaction mixtures, however, only in two cases we managed to isolate the latter compounds in individual state. At first we decided to verify the efficiency of the catalytic system with **L2** and Ph_3_P and tried it in the reaction with 4-iodotoluene, but the conversion of the diamine into the diarylated product **18** was low (Table 2, entry 6). Only using 20/40 mol % CuI/**L2** we managed to obtain the desired compound in a good yield (Table 2, entry 7). The same catalytic system with or without triphenylphosphine was not efficient in the coupling with 4-iodobiphenyl, however, the use of **L1** gave rise to the target diaryl derivative **19** in a moderate yield (Table 2, entry 8). On contrary, application of the ligand **L2** was more successful for aryl iodides with electron-withdrawing groups like Cl, F and CF_3_, and the best results were obtained in the presence of Ph_3_P (Table 2, entries 10–13), the yields of compounds **20**, **22** and **23** ranged from 52 to 69%. The reaction with 4-iodoanisole catalyzed with CuI/**L2**/Ph_3_P provided 56% yield of the monoaryl derivative **24** (Table 2, entry 14); we tried other catalytic systems but they worked even worse providing low conversion of starting compounds. It should be noted that in the case of 4-chloro- and 4-fluoroiodobenzene the use of 20 mol % catalyst provided higher conversion of the starting compounds and better yields, but in the case of other aryl iodides 20 mol % catalyst led to side processes diminishing the yields of the target compounds.

**Scheme 3:**
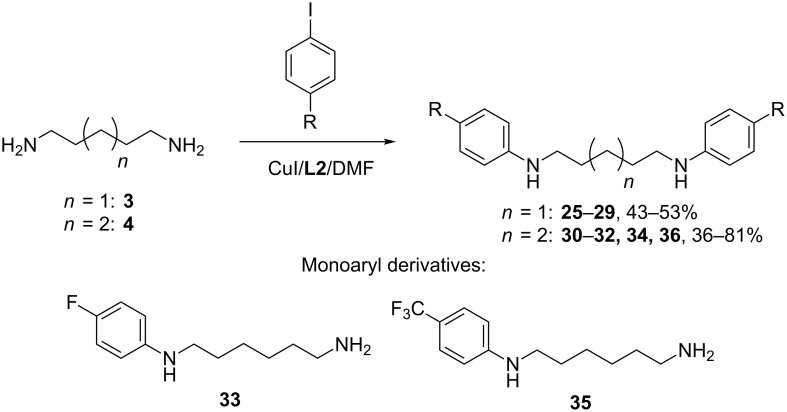
Arylation of the diamines **3** and **4**.

Contrary to the diarylation of putrescine, the same processes with cadaverine (**3**) and hexane-1,6-diamine (**4**) proceeded without serious difficulties (Scheme 3, Table 3). The reactions of the diamine **3** with iodobenzene and other *para*-disubstituted benzenes gave the desired products in 43–53% yields in the presence of the CuI/**L2** catalytic system (Table 3, entries 1–4). Only the application of 4-iodoanisole under stated conditions gave diaryl derivative **29** in a rather poor yield (Table 3, entries 5 and 6), but the use of the ligand **L1** improved the result (Table 3, entry 7). In the case of diamine **4** the yields of the corresponding products **30–32** and **34** were somewhat lower (Table 3, entries 8–11), and with fluorinated compounds we managed to isolate also monoarylated derivatives **33** and **35** (Table 3, entries 10 and 11). It is interesting that 20/40 mol % CuI/**L2** catalytic system provided a high yield (81%) of the *N,N’*-diarylation product in the reaction with 4-iodoanisole (Table 3, entry 12). It shows that in some cases an increase in the catalyst loading can substantially enhance the yields in the reactions even with less reactive aryl halides.

**Table 3:** Arylation of the diamines **3** and **4**.

Entry	Amine	R	CuI/**L2**, mol %	Products and yields, %^a^

1	**3**	H	10/20	**25**, 50
2	**3**	Ph	10/20	**26**, 43
3	**3**	F	10/20	**27**, 53
4	**3**	CF_3_	10/20	**28**, 51
5	**3**	OMe	10/20	**29**, 22
6	**3**	OMe	20/40	**29**, 22
7	**3**	OMe	20/40^b^	**29**, 52
8	**4**	H	10/20	**30**, 45
9	**4**	Ph	10/20	**31**, 36
10	**4**	F	10/20	**32**, 38; **33**, 58
11	**4**	CF_3_	10/20	**34**, 34; **35**, 12
12	**4**	OMe	20/40	**36**, 81

^a^Yields after chromatographic isolation. ^b^Ligand **L1** was used.

**Scheme 4:**
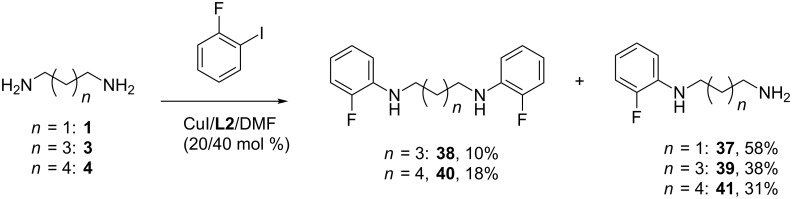
Arylation of the diamines **1**, **3**, **4** with 2-fluoroiodobenzene.

Cu(I)-catalyzed amination of *ortho*-disubstituted benzenes is a challenging task as earlier we demonstrated very low reactivity of 2-iodotoluene and 1,2-diiodobenzene [31]. Contrary to this, Pd(0)-catalyzed amination of 2-bromotoluene derivatives was quite successful [36]. In this study we tried the reaction of 2-fluoroiodobenzene with diamines **1**, **3** and **4** using the CuI/**L2** catalytic system (20/40 mol %) (Scheme 4). In the reaction with propane-1,3-diamine (**1**) only monoaryl derivative **37** was obtained in 58% yield, and with diamines **3** and **4** we managed to isolate *N,N’*-diaryl derivatives **38** and **40**, though their yields were too small (10 and 18%, respectively), the main products being *N*-(2-fluorophenyl) diamines **39** and **41**.

### *N,N’*-Diarylation of tri- and tetraamines

**Scheme 5:**
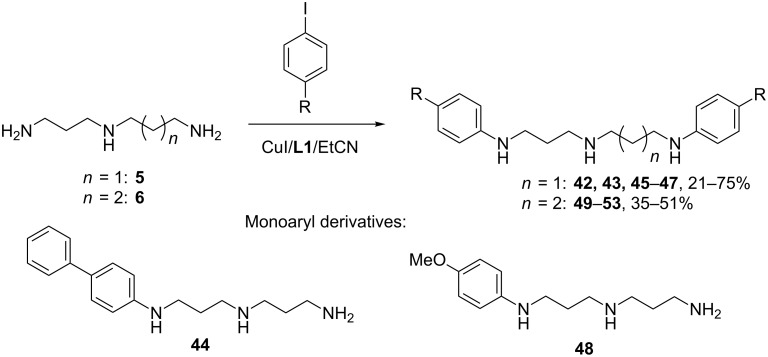
Arylation of the triamines **5** and **6**.

Arylation of two primary amino groups in polyamines under Cu(I)-catalysis conditions is a more challenging task than *N*,*N*’-diarylation of diamines because copper-catalyzed amination is less selective than palladium-catalyzed coupling. In view of earlier obtained data we used the CuI/**L1**/EtCN catalytic system for the diarylation of the triamine **5** (Scheme 5, Table 4).

**Table 4:** Arylation of the triamines **5** and **6**.

Entry	Amine	R	CuI/**L1**, mol %	Products and yields, %^a^

1	**5**	H	10/20	**42**, 41^b^
2	**5**	H	10/20^b^	
3	**5**	Ph	10/20	**43**, 13; **44**, 18
4	**5**	Ph	20/40	**43**, 75
5	**5**	F	10/20	**45**, 50^b^
6	**5**	F	20/40	
7	**5**	CF_3_	10/20	**46**, 53
8	**5**	OMe	20/40	**47**, 21; **48**, 35
9	**6**	H	10/20	**49**, 36
10	**6**	Ph	10/20	**50**, 24
11	**6**	Ph	20/40	**50**, 35
12	**6**	F	10/20	**51**, 35
13	**6**	CF_3_	10/20	**52**, 51
14	**6**	OMe	10/20^c^	**53**, 27
15	**6**	OMe	20/40^c^	**53**, 46

^a^Yields after chromatographic isolation. ^b^DMF was used instead of EtCN, chromatography of combined reaction mixtures. ^c^Ligand **L2** was used.

In all cases this catalytic system was efficient and corresponding *N,N’*-diaryl derivatives **42**, **45**, **46** were obtained in satisfactory yields (41–53%, Table 4, entries 1, 5 and 7) when taking 10/20 mol % of catalyst, and in the reaction with 4-iodobiphenyl 20/40 mol % catalyst allowed to increase the yield of compound **43** to 75% (Table 4, entry 4) and to obtain the diarylation product **47** with 4-iodoanisole, though in a small amount (Table 4, entry 8). The application of the CuI/**L1** system in DMF gave the same results, as the NMR spectra of the reaction mixture revealed, thus the chromatographic isolation of the combined reaction mixtures was carried out (Table 4, entries 1 and 2, 5 and 6). However, the CuI/**L2**/DMF system was not efficient at all as it strongly diminished the selectivity of the reactions.

Unsymmetrical spermidine (**6**) provided somewhat poorer yields of the diarylated derivatives **49**–**52** (Table 4, entries 9–13), and to obtain the diarylation product with the 4-iodoanisole CuI/**L2**/DMF catalytic system had to be used (Table 4, entries 14 and 15). The application of 20 mol % catalyst instead of 10 mol % helped to increase the yields in some cases (entries 11 and 15).

**Scheme 6:**
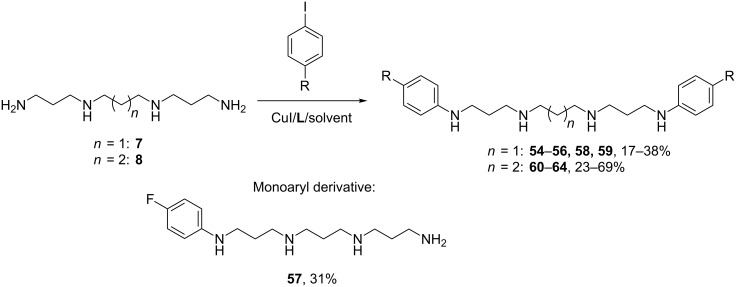
Arylation of the tetraamines **7** and **8**.

Easier arylation of the secondary amino groups in the tetraamines **7** and **8** (Scheme 6, Table 5) led to a loss in the selectivity of the process, made chromatographic isolation more tedious and less efficient, and also diminished the catalytic activity of copper due to better coordination of the cation by four nitrogen atoms which removed it from the catalytic cycle.

**Table 5:** Arylation of the tetraamines **7** and **8**.

Entry	Amine	R	Catalytic system	CuI/L, mol %	Products and yields, %^a^

1	**7**	H	CuI/**L1**/EtCN	10/20	**54**, 37
2	**7**	H	CuI/**L2**/DMF	10/20	**54**, 24
3	**7**	Ph^b^	CuI/**L1**/EtCN	14/28	**55**, 32
4	**7**	F	CuI/**L1**/EtCN	10/20	**56**, 6; **57**, 31
5	**7**	F	CuI/**L2**/Ph_3_P/DMF	10/10/10	**56**, 37
6	**7**	CF_3_	CuI/**L1**/EtCN	10/20	**58**, 38
7	**7**	CF_3_	CuI/**L2**/DMF	10/20	**58**, 8
8	**7**	OMe	CuI/**L2**/DMF	20/40	**59**, 8
9	**7**	OMe^c^	CuI/**L2**/DMF	20/40	**59**, 17
10	**8**	H	CuI/**L2**/DMF	10/20	**60**, 69
11	**8**	Ph	CuI/**L1**/EtCN	10/20	**61**, 36
12	**8**	F	CuI/**L2**/DMF	10/20	**62**, 45
13	**8**	CF_3_	CuI/**L2**/DMF	10/20	**63**, 41
14	**8**	OMe	CuI/**L2**/DMF	20/40	**64**, 23

^a^Yields after chromatographic isolation. ^b^3.4 equiv 4-iodobiphenyl were used. ^c^10 equiv 4-iodoanisole were used.

The *N,N’*-diarylation of the tetraamine **7** in some cases was more successful in the presence of the CuI/**L1**/EtCN catalytic system (Table 5, entries 1, 3 and 6) while CuI/**L2**/DMF gave bad results due to low conversion of the starting compounds or poor selectivity of the arylation. On contrary, the latter system was found to be more appropriate in the case of 4-iodoanisole, though the yield of the target product **59** was low even when using 10 equiv of the arylating agent (Table 5, entry 9). In the reaction with 4-fluoroiodobenzene the target compound **56** could be obtained only in the presence of the CuI/**L2**/Ph_3_P system (Table 5, entry 5). The reactions with spermine (**8**) which possesses tetramethylenediamine central fragment gave better yields. In the majority of cases the system CuI/**L2**/DMF was more efficient (Table 5, entries 10, 12 and 13), CuI/**L1**/EtCN provided insufficient conversion of the starting compounds, and only with 4-iodobiphenyl it was more helpful (Table 5, entry 11). The reaction with 4-iodoanisole was the most problematic and the yield of **64** even with 20 mol % of catalyst did not exceed 23% (Table 5, entry 14).

## Conclusion

To sum up, the following regularities can be ruled out from the experiments: a) successful N,N’-diarylation of the diamines **1**, **3**, **4** and spermine (**8**) can be carried out in the presence of the CuI/**L2**/DMF catalytic system, while triamines **5**, **6** and norspermine (**7**) prefer the CuI/**L1**/EtCN system; b) the most problematic amines are putrescine (**2**) and norspermine (**7**) as they demand a fine tuning of the catalytic system almost for each aryl iodide; c) compounds with electron-withdrawing substituents (Cl, F, CF_3_) generally produce *N*,*N*’-diarylated derivatives in reasonable yields, while the reactivity of electron-enriched 4-iodoanisole is lower in many cases and more catalyst is needed to afford diarylation products.

## Supporting Information

File 1 Experimental procedures, characterization and spectral data for synthesized compounds **11**–**64**.
