# Supplementary material for: Cu(I)-catalyzed N,N’-diarylation of natural diamines and polyamines with aryl iodides
Source: Beilstein J Org Chem. 2015 Nov 24;11:2297–305. doi: 10.3762/bjoc.11.250 (PMC4685767; doi:10.3762/bjoc.11.250)
Supplement: File 1 — Experimental procedures, characterization and spectral data for synthesized compounds 11–64. [file Beilstein_J_Org_Chem-11-2297-s001.pdf]

Supporting Information

for

Cu(I)-catalyzed *N,N'*-diarylation of natural diamines and polyamines with aryl iodides

Svetlana P. Panchenko, Alexei D. Averin, Maksim V. Anokhin, Olga A. Maloshitskaya and Irina P. Beletskaya*

Address: Department of Chemistry, Lomonosov Moscow State University, Leninskie Gory 1-3, Moscow, 119991, Russia

Email: Irina P. Beletskaya* - beletska@org.chem.msu.ru

*Corresponding author

Experimental procedures, characterization and spectral data for synthesized compounds 11–64

General

NMR spectra were recorded with a Bruker Avanve-400 spectrometer (^{1}H at 400 MHz, ^{13}C at 100.6 MHz) in $CDCl_3$ or DMSO-d_6 at 298 K. Chemical shifts are given in δ scale in ppm, signals in proton spectra are referenced to residual peaks of $CHCl_3$ or CHD_2 in DMSO (δ_H 7.25, 2.49, respectively), signals in ^{13}C spectra are referenced to the centers of multiplets $CDCl_3$ or $(CD_3)_2SO$ (δ_c 77.0, 39.5, respectively). MALDI-TOF mass spectra of positive ions were registered with a Bruker Daltonics Autoflex II spectrometer using 1,8,9-trihydroxyanthracene (dithranol) as a matrix and PEG-200, 300, 400 or 600 as internal standards for a precise calibration. Elemental analysis was done using Vario Micro Cube Elementar device, melting points were measured with Electrothermal device in a open capillary. Preparative column chromatography was carried out using silica gel Merck 40–60 mesh.

Commercially available diamines and polyamines **1–8**, copper iodide, cesium carbonate, 2-(isobutyryl)cyclohexanone, *L*-proline and aryl iodides were used without special purification. Dimethylformamide and propionitrile were distilled over CaH_2, dichloromethane, petroleum ether and methanol were used freshly distilled.

General method for Cu(I)-catalyzed *N,N'*-diarylation of di- and polyamines

A Schlenk tube equipped with a magnetic stirrer and reflux condenser, flushed with dry argon, was charged with CuI (10-20 mol %), ligand (L-proline (**L1**) or 2-(isobutyryl)cyclohexanone (**L2**), 20-40 mol %), aryl iodide (2.5 mmol), appropriate solvent (DMF or EtCN, 2 mL), and the di- or polyamine (1 mmol). The reaction mixture was stirred for 1 min, then cesium carbonate (2.5 mmol, 845 mg) was added and the reaction was stirred either under reflux (in the case of EtCN) or at 110 °C (in the case of DMF). After ca 24 h upon completion of the reaction the mixture was cooled down to ambient temperature, dichloromethane (5 mL) was added, the organic solution was filtered, the residue was washed additionally with dichloromethane (2 × 5 mL), the combined organic fractions were evaporated in vacuo. To obtain individual compounds, the residue was chromatographed on silica gel using a sequence of eluents: CH_2Cl_2, CH_2Cl_2/MeOH (10:1, 3:1), CH_2Cl_2/MeOH/NH_3(aq) (100:20:1, 100:20:2, 100:20:3, 100:25:5, 10:4:1) or hexanes/CH_2Cl_2 (5:1, 3:1, 2:1, 1:1, 1:2), CH_2Cl_2, CH_2Cl_2/MeOH (100:1).

N^1,N^3-diphenylpropane-1,3-diamine (**9**), N^1-phenylpropane-1,3-diamine (**10**), N^1-phenylbutane-1,4-diamine (**12**) are described in literature (ref. [1-3]) and their yields were estimated in the reaction mixtures from ^{1}H and ^{13}C NMR spectra. These reactions were used for preliminary experiments to compare efficiency of the catalytic systems.

N^1,N^4-Diphenylbutane-1,4-diamine (**11**) [1] was obtained from diamine **2** (1 mmol, 101 μL), iodobenzene (2.5 mmol, 280 μL) in the presence of CuI (19 mg) and L-proline (23 mg) in 2 mL EtCN. Eluent hexanes/CH_2Cl_2 1:1-1:2. Yield 103 mg (43%), light-yellow viscous oil. ^{1}H NMR ($CDCl_3$) δ 1.72-1.78 (m, 4H, C**CH_2CH_2**C), 3.18 (t, 4H, ^3J = 5.6 Hz, CH_2N), 3.54 (br.s, 2H, NH), 6.62 (d, 4H, $^3J_{obs}$ = 7.9 Hz, H2, H2'(Ph)), 6.73 (t, 2H, $^3J_{obs}$ = 7.3 Hz, H4(Ph)), 7.20 (t, 4H, $^3J_{obs}$ = 7.9 Hz, H3, H3'(Ph)); ^{13}C NMR ($CDCl_3$) δ 27.1 (2C, C**CH_2CH_2**C), 43.7 (2C, CH_2N), 112.7 (4C, C2, C2'(Ph)), 117.3 (2C, C4(Ph)), 129.2 (4C, C3, C3'(Ph)), 148.2 (2C, C1(Ph)); MS (MALDI-TOF) calcd. for $C_{16}H_{21}N_2$: 241.17, found 241.16 $[M+H]^+$.

N^1-Phenylbutane-1,4-diamine (**12**) [4] was obtained as the second product in the synthesis of compound **11** and was identified in the reaction mixture by proton NMR spectroscopy. ^{1}H NMR ($CDCl_3$) δ 1.50-1.58 (m, 2H, C**CH_2**CH_2C), 1.60-1.68 (m, 2H, CCH_2**CH_2**C), 2.73 (br.s, 2H, **CH_2**NH_2), 3.12 (m, 2H, ^3J = 6.9 Hz, CH_2NPh), 6.59 (d, 2H, $^3J_{obs}$ = 8.0 Hz, H2, H2'(Ph)), 6.69 (t, 1H, ^3J = 7.2 Hz, H4(Ph)), 7.13-7.20 (m, 2H, H3, H3'(Ph)), NH and NH_2 protons were not unambiguously assigned.

***N*[1],*N*[3]-Di(biphenyl-4-yl)propane-1,3-diamine** (**13**) [5] was obtained from diamine **1** (1 mmol, 83 μL), 4-iodobiphenyl (2.5 mmol, 700 mg) in the presence of CuI (19 mg) and 2-(isobutyryl)cyclohexanone (33 μL) in 2 mL DMF. Eluent CH_2Cl_2 – CH_2Cl_2/MeOH 100:1. Yield 211 mg (56%), pale-brown crystalline powder, m.p. 118-120°C. ^{1}H NMR ($CDCl_3$) δ 1.98 (quintet, 2H, ^3J = 6.7 Hz, C**CH_2**C), 3.33 (*t,* 4H, ^{3}J = 6.7 Hz, CH_2NPh), 3.76 (br.s, 2H, NH), 6.73 (d, 4H, $^3J_{obs}$ = 8.6 Hz, H(Ar)), 7.32 (t, 2H, ^3J = 7.3 Hz, H(Ar)), 7.45 (t, 4H, $^3J_{obs}$ = 7.6 Hz, H(Ar)), 7.51 (d, 4H, $^3J_{obs}$ = 8.6 Hz, H(Ar)), 7.58-7.62 (m, 4H, H(Ar)); ^{13}C NMR ($CDCl_3$) δ 29.1 (1C, C**CH_2**C), 41.9 (2C, CH_2NPh), 113.1 (4C, CH(Ar)), 126.0 (2C, CH(Ar)), 126.2 (4C, CH(Ar)), 127.9 (4C, CH(Ar)), 128.6 (4C, CH(Ar)), 130.3 (2C, C(Ar)), 141.1 (2C, C(Ar)), 147.5 (2C, NC(Ar)). MS (MALDI-TOF) calcd. for $C_{27}H_{27}N_2$: 379.22, found 379.24 $[M+H]^+$.

***N*[1],*N*[3]-Bis(4-chlorophenyl)propane-1,3-diamine** (**14**) was obtained from diamine **1** (1 mmol, 83 μL), 4-chloroiodobenzene (2.5 mmol, 596 mg) in the presence of CuI (19 mg) and 2-(isobutyryl)cyclohexanone (33 μL) in 2 mL DMF. Eluent CH_2Cl_2/MeOH 100:1. Yield 209 mg (71%), yellow crystalline powder, m.p. 85-87°C. ^{1}H NMR ($CDCl_3$) δ 1.89 (quintet, 2H, ^3J = 6.7 Hz, C**CH_2**C), 3.20 (t, 4H, ^3J = 6.7 Hz, CH_2NPh), 3.72 (br.s, 2H, NH), 6.50-6.54 (m, 4H, H2, H2'(Ph)), 7.10-7.14 (m, 4H, H3, H3'(Ph)); ^{13}C NMR ($CDCl_3$) δ 28.9 (1C, C**CH_2**C), 41.9 (2C, CH_2NPh), 113.8 (4C, C2, C2'(Ph)), 121.9 (2C, C4(Ph)), 129.0 (4C, C3, C3'(Ph)), 146.6 (2C, C1(Ph)); HRMS (MALDI-TOF) calcd. for $C_{15}H_{17}Cl_2N_2$: 295.0769, found 295.0784 $[M+H]^+$.

***N*[1],*N*[3]-Bis(4-fluorophenyl)propane-1,3-diamine** (**15**) was obtained from diamine **1** (1 mmol, 83 μL), 4-fluoroiodobenzene (2.5 mmol, 288 μL) in the presence of CuI (19 mg) and 2-(isobutyryl)cyclohexanone (33 μL) in 2 mL DMF. Eluent CH_2Cl_2/MeOH 50:1-25:1. Yield 160 mg (61%), yellow viscous oil. ^{1}H NMR ($CDCl_3$) δ 1.92 (quintet, 2H, ^3J = 6.7 Hz, C**CH_2**C), 3.21 (t, 4H, ^3J = 6.7 Hz, CH_2NPh), 3.61 (br.s, 2H, NH), 6.53-6.57 (m, 4H, $^4J_{HF}$ = 4.3 Hz, H2, H2'(Ph)), 6.86-6.92 (m, 4H, $^3J_{HF}$ = 8.6 Hz, H3, H3'(Ph)); ^{13}C NMR ($CDCl_3$) δ 29.1 (1C, C**CH_2**C), 42.7 (2C, CH_2NPh), 113.6 (d, 4C, $^3J_{CF}$ = 6.8 Hz, C2, C2'(Ph)), 115.6 (d, 4C, $^2J_{CF}$ = 21.9 Hz, C3, C3'(Ph)), 144.5 (2C, C1(Ph)), 155.8 (d, 2C, $^1J_{CF}$ = 235.2 Hz, C4(Ph)); HRMS (MALDI-TOF) calcd. for $C_{15}H_{17}F_2N_2$: 263.1360, found 263.1429 $[M+H]^+$.

***N*[1],*N*[3]-Bis(4-(trifluoromethyl)phenyl)propane-1,3-diamine** (**16**) was obtained from diamine **1** (1 mmol, 83 μL), 4-iodo(trifluoromethyl)benzene (2.5 mmol, 367 μL) in the presence of CuI (19 mg) and 2-(isobutyryl)cyclohexanone (33 μL) in 2 mL DMF. Eluent hexanes/CH_2Cl_2 1:1. Yield

147 mg (42%), pale-yellow crystalline powder, m.p. 96-98°C. ^{1}H NMR ($CDCl_3$) δ 1.96 (quintet, 2H, ^{3}J = 6.7 Hz, C**CH₂**C), 3.30 (t, 4H, ^{3}J = 6.7 Hz, CH_2NPh), 4.03 (br.s, 2H, NH), 6.61 (d, 4H, $^{3}J_{obs}$ = 8.5 Hz, H2, H2'(Ph)), 7.41 (d, 4H, $^{3}J_{obs}$ = 8.5 Hz, H3, H3'(Ph)); ^{13}C NMR ($CDCl_3$) δ 28.8 (1C, C**CH_2**C), 41.3 (2C, CH_2NPh), 111.9 (4C, C2, C2'(Ph)), 118.9 (q, 2C, $^{2}J_{CF}$ = 33.2 Hz, C4(Ph)), 125.0 (q, 2C, $^{1}J_{CF}$ = 270.0 Hz, CF_3), 126.7 (4C, C3, C3'(Ph)), 150.5 (2C, C1(Ph)); HRMS (MALDI-TOF) calcd. for $C_{17}H_{17}F_6N_2$: 363.1296, found 363.1328 $[M+H]^+$.

***N*^{1},*N*^{3}-Bis(4-methoxyphenyl)propane-1,3-diamine** (**17**) was obtained from diamine **1** (1 mmol, 83 μL), 4-iodoanisole (2.5 mmol, 585 mg) in the presence of CuI (19 mg) and 2-(isobutyryl)cyclohexanone (33 μL) in 2 mL DMF. Eluent CH_2Cl_2/MeOH 25:1. Yield 159 mg (56%), brown crystalline powder, m.p. 81-83°C. ^{1}H NMR ($CDCl_3$) δ 1.89 (quintet, 2H, ^{3}J = 6.4 Hz, C**CH_2**C), 3.19 (br.s, 4H, CH_2NPh), 3.73 (s, 6H, OCH_3), 6.57 (d, 4H, $^{3}J_{obs}$ = 8.7 Hz, H2, H2'(Ph)), 6.77 (d, 4H, $^{3}J_{obs}$ = 8.7 Hz, H3, H3'(Ph)), NH protons were not unambiguously assigned; ^{13}C NMR ($CDCl_3$) δ 29.3 (1C, C**CH_2**C), 43.0 (2C, CH_2NPh), 55.7 (2C, OCH_3), 114.1 (4C, CH(Ph)), 114.8 (4C, CH(Ph)), 142.4 (2C, C1(Ph)), 152.1 (2C, C4(Ph)); HRMS (MALDI-TOF) calcd. for $C_{17}H_{23}N_2O_2$: 287.1760, found 287.1725 $[M+H]^+$.

***N*^{1},*N*^{4}-Di-*p*-tolylbutane-1,4-diamine** (**18**) was obtained from diamine **2** (1 mmol, 101 μL), 4-iodotoluene (2.5 mmol, 545 mg) in the presence of CuI (38 mg) and 2-(isobutyryl)cyclohexanone (67 μL) in 2 mL DMF. Eluent CH_2Cl_2 – CH_2Cl_2/MeOH 50:1. Yield 160 mg (60%), beige crystalline powder, m.p. 79-81°C. ^{1}H NMR ($CDCl_3$) δ 1.69-1.75 (m, 4H, C**CH_2CH_2**C), 2.26 (s, 6H, CH_3), 3.12-3.18 (m, 4H, CH_2NPh), 3.51 (br.s, 2H, NH), 6.55 (d, 4H, $^{3}J_{obs}$ = 8.3 Hz, H2, H2'(Ph)), 7.01 (d, 4H, $^{3}J_{obs}$ = 8.3 Hz, H3, H3'(Ph)); ^{13}C NMR ($CDCl_3$) δ 20.4 (2C, CH_3), 27.1 (2C, C**CH_2CH_2**C), 44.3 (2C, CH_2NPh), 113.2 (4C, C2, C2'(Ph)), 126.8 (2C, C4(Ph)), 129.7 (4C, C3, C3'(Ph)), 145.8 (2C, C1(Ph)); HRMS (MALDI-TOF) calcd. for $C_{18}H_{25}N_2$: 269.2018, found 269.1983 $[M+H]^+$.

***N*^{1},*N*^{4}-Di(biphenyl-4-yl)butane-1,4-diamine** (**19**) was obtained from diamine **2** (1 mmol, 101 μL), 4-iodobiphenyl (2.5 mmol, 700 mg) in the presence of CuI (19 mg) and L-proline (23 mg) in 2 mL EtCN. Eluent CH_2Cl_2. Yield 106 mg (46%), pale-yellow viscous oil. ^{1}H NMR ($CDCl_3$) δ 1.78 (br.s, 4H, C**CH_2CH_2**C), 3.23 (br.s, 4H, CH_2N), 3.79 (br.s, 2H, NH), 6.68 (d, 4H, $^{3}J_{obs}$ = 8.5 Hz, H(Ph)), 7.26 (t, 2H, ^{3}J = 7.3 Hz, H(Ph)), 7.39 (t, 4H, $^{3}J_{obs}$ = 7.7 Hz, H(Ph)), 7.45 (d, 4H, $^{3}J_{obs}$ = 8.5 Hz, H(Ph)), 7.54 (d, 4H, $^{3}J_{obs}$ = 7.3 Hz, H(Ph)); ^{13}C NMR ($CDCl_3$) δ 27.1 (2C, C**CH_2CH_2**C), 43.7 (2C, CH_2N), 113.0 (4C, CH(Ph)), 126.0 (2C, CH(Ph)), 126.3 (4C, CH(Ph)),

128.0 (4C, CH(Ph)), 128.6 (4C, CH(Ph)), 130.3 (2C, C(Ph)), 141.2 (2C, C(Ph)), 147.6 (2C, NC(Ph)); HRMS (MALDI-TOF) calcd. for $C_{28}H_{29}N_2$: 393.2331, found 393.2367 $[M+H]^+$. By the treatment with 5 M HCl solution in dioxane corresponding dihydrochloride was obtained as pale-beige crystalline powder, m.p. 210°C (decomp.). Calcd. for $C_{28}H_{30}Cl_2N_2$ (%):C 72.25, H 6.50, N 6.02; found C 72.01, H 6.59, N 5.83.

***N*^{1},*N*^{4}-Bis(4-chlorophenyl)butane-1,4-diamine** (**20**) was obtained from diamine **2** (1 mmol, 101 μL), 4-chloroiodobenzene (2.5 mmol, 596 mg) in the presence of CuI (38 mg), Ph_3P (52 mg) and 2-(isobutyryl)cyclohexanone (33 μL) in 2 mL DMF. Eluent hexanes/CH_2Cl_2 2:1-1:4. Yield 212 mg (69%), beige crystalline powder, m.p. 108-110°C. ^{1}H NMR ($CDCl_3$) δ 1.68-1.74 (m, 4H, C**CH$_2$CH$_2$**C), 3.09-3.15 (m, 4H, CH_2N), 3.63 (br.s, 2H, NH), 6.50 (d, 4H, $^3J_{obs}$ = 8.6 Hz, H2, H2'(Ph)), 7.11 (d, 4H, $^3J_{obs}$ = 8.6 Hz, H3, H3'(Ph)); ^{13}C NMR ($CDCl_3$) δ 26.9 (2C, C**CH$_2$CH$_2$**C), 43.7 (2C, CH_2N), 113.7 (4C, C2, C2'(Ph)), 121.8 (2C, C4(Ph)), 129.0 (4C, C3, C3'(Ph)), 146.7 (2C, C1(Ph)); HRMS (MALDI-TOF) calcd. for $C_{16}H_{19}Cl_2N_2$: 309.0925, found 309.0901 $[M+H]^+$. When carrying out the same reaction in the presence of CuI (19 mg), Ph_3P (26 mg) and 2-(isobutyryl)cyclohexanone (17 μl), the yield of compound **20** was 83 mg (27%).

***N*^{1}-(4-Chlorophenyl)butane-1,4-diamine** (**21**) [4] was obtained as the second product in the synthesis of compound **20** in the presence of CuI (19 mg), Ph_3P (26 mg) and 2-(isobutyryl)cyclohexanone (17 μL). Eluent CH_2Cl_2 – CH_2Cl_2/MeOH 5:1. Yield 69 mg (35%), brown viscous oil. ^{1}H NMR ($CDCl_3$) δ 1.51-1.64 (m, 4H, C**CH$_2$CH$_2$**C), 2.80 (t, 2H, ^3J = 7.1 Hz, **CH$_2$**NH$_2$), 2.98 (q, 2H, ^3J = 5.6 Hz, CH_2NPh), 5.79 (t, 2H, ^3J = 4.9 Hz, NHPh), 6.55 (d, 2H, $^3J_{obs}$ = 8.7 Hz, H2, H2'(Ph)), 7.05 (d, 2H, $^3J_{obs}$ = 8.7 Hz, H3, H3'(Ph)) NH_2 protons were not unambiguously assigned; ^{13}C NMR ($CDCl_3$) δ 24.7 (1C, C**CH$_2$**CH$_2$C), 25.4 (1C, CCH$_2$**CH$_2$**C), 38.7 (1C, CH_2NH_2), 42.2 (1C, CH_2NPh), 113.2 (2C, C2, C2'(Ph)), 118.6 (1C, C4(Ph)), 128.5 (2C, C3, C3'(Ph)), 147.7 (1C, C1(Ph)). MS (MALDI-TOF) calcd. for $C_{10}H_{16}ClN_2$: 199.10, found 199.08 $[M+H]^+$.

***N*^{1},*N*^{4}-Bis(4-fluorophenyl)butane-1,4-diamine** (**22**) was obtained from diamine **2** (1 mmol, 101 μL), 4-fluoroiodobenzene (2.5 mmol, 288 μL) in the presence of CuI (38 mg), Ph_3P (52 mg) and 2-(isobutyryl)cyclohexanone (33 μL) in 2 mL DMF. Eluent hexanes/CH_2Cl_2 1:2 – CH_2Cl_2. Yield 144 mg (52%), pale-yellow viscous oil. ^{1}H NMR ($CDCl_3$) δ 1.72 (br.s, 4H, C**CH$_2$CH$_2$**C), 3.11 (br.s, 4H, CH_2N), 3.42 (br.s, 2H, NH), 6.53 (dd, 4H, $^3J_{HHobs}$ = 8.5 Hz, $^4J_{HF}$ = 4.5 Hz, H2, H2'(Ph)), 6.88 (dd, 4H, $^3J_{HHobs}$ = 8.5 Hz, $^3J_{HF}$ = 8.5 Hz, H3, H3'(Ph)); ^{13}C NMR (DMSO-d_6)

δ 23.1 (2C, C**CH$_2$CH$_2$**C), 48.5 (2C, CH$_2$N), 116.4 (d, 4C, $^2J_{CF}$ = 22.7 Hz, C3, C3'(Ph)), 122.8 (br.s, 4C, C2, C2'(Ph)), 135.3 (2C, C1(Ph)), C4(Ph) quaternary carbon atom was not unambiguously assigned due to very low intensity caused by line broadening; HRMS (MALDI-TOF) calcd. for $C_{16}H_{19}F_2N_2$: 277.1516, found 277.1490 $[M+H]^+$.

***N*^{1},*N*^{4}-Bis(4-(trifluoromethyl)phenyl)butane-1,4-diamine** (**23**) was obtained from diamine **2** (1 mmol, 101 μL), 4-iodo(trifluoromethyl)benzene (2.5 mmol, 367 μL) in the presence of CuI (19 mg), Ph_3P (26 mg) and 2-(isobutyryl)cyclohexanone (17 μL) in 2 mL DMF. Eluent hexanes/CH_2Cl_2 3:1-1:1. Yield 216 mg (58%), pale-yellow viscous oil. ^{1}H NMR ($CDCl_3$) δ 1.72-1.77 (m, 4H, C**CH$_2$CH$_2$**C), 3.17-3.22 (m, 4H, CH$_2$N), 4.01 (br.s, 2H, NH), 6.58 (d, 4H, $^3J_{obs}$ = 8.5 Hz, H2, H2'(Ph)), 7.40 (d, 4H, $^3J_{obs}$ = 8.5 Hz, H3, H3'(Ph)); ^{13}C NMR ($CDCl_3$) δ 26.8 (2C, C**CH$_2$CH$_2$**C), 43.2 (2C, CH$_2$N), 111.8 (4C, C2, C2'(Ph)), 118.8 (q, 2C, $^2J_{CF}$ = 32.9 Hz, C4(Ph)), 125.0 (q, 2C, $^1J_{CF}$ = 270.6 Hz, CF$_3$), 126.6 (4C, C3, C3'(Ph)), 150.5 (2C, C1(Ph)); HRMS (MALDI-TOF) calcd. for $C_{18}H_{19}F_6N_2$: 377.1452, found 377.1421 $[M+H]^+$; calcd. for $C_{18}H_{18}F_5N_2$: 357.1390, found 357.1362 $[M-F]^+$. By the treatment with 5M HCl solution in dioxane corresponding hydrochloride as a solvate with dioxane was obtained as pale-beige crystalline powder, m.p. 163-165°C. Calcd. for $C_{18}H_{19}ClF_6N_2{*}C_4H_8O$ (%):C 52.75, H 5.43, N 5.59; found C 53.02, H 5.95, N 5.96.

***N*^{1}-(4-Methoxyphenyl)butane-1,4-diamine** ((**24**) [4] was obtained from diamine **2** (1 mmol, 101 μL), 4-iodoanisole (2.5 mmol, 585 mg) in the presence of CuI (19 mg), Ph_3P (26 mg) and 2-(isobutyryl)cyclohexanone (17 μL) in 2 mL DMF. Eluent hexanes/CH_2Cl_2 3:1. Yield 112 mg (56%), brown viscous oil. ^{1}H NMR ($CDCl_3$) δ 1.64 (br.s, 4H, C**CH$_2$CH$_2$**C), 2.81 (br.s, 2H, **CH$_2$**NH$_2$), 3.04 (br.s, 2H, C**H$_2$**NHPh), 3.71 (s, 3H, CH$_3$O), 4.14 (br.s, 3H, NH, NH$_2$), 6.61 (d, 4H, $^3J_{obs}$ = 8.7 Hz, H2, H2'(Ph)), 6.75 (d, 4H, $^3J_{obs}$ = 8.7 Hz, H3, H3'(Ph)); ^{13}C NMR ($CDCl_3$) δ 26.8 (1C, C**CH$_2$**CH$_2$C), 28.8 (1C, CCH$_2$**CH$_2$**C), 41.0 (1C, CH$_2$NH$_2$), 44.8 (1C, CH$_2$NHPh), 55.8 (1C, CH$_3$O), 114.6 (2C, CH(Ph)), 114.9 (2C, CH(Ph)), 142.3 (1C, C1(Ph)), 152.4 (1C, C4(Ph)). MS (MALDI-TOF) calcd. for $C_{11}H_{19}N_2O$: 195.15, found 195.18 $[M+H]^+$

***N*^{1},*N*^{5}-Diphenylpentane-1,5-diamine** (**25**) [6] was obtained from diamine **3** (1 mmol, 118 μl), iodobenzene (2.5 mmol, 280 μL) in the presence of CuI (19 mg) and 2-(isobutyryl)cyclohexanone (33 μL) in 2 mL DMF. Eluent hexanes/CH_2Cl_2 1:2. Yield 127 mg (50%), pale-yellow viscous oil. ^{1}H NMR ($CDCl_3$) δ 1.53-1.62 (m, 2H, NCCCH$_2$), 1.73 (quintet, 4H, $^3J_{obs}$ = 7.4 Hz, NCCH$_2$), 3.19 (t, 4H, ^3J = 7.1 Hz, CH$_2$N), 3.63 (br.s, 2H, NH), 6.68 (dd, 4H,

$^{3}J_{obs}$ = 8.5 Hz, ^{4}J = 0.9 Hz, H2, H2'(Ph)), 6.79 (tt, 2H, ^{3}J = 7.3 Hz, ^{4}J = 0.9 Hz, H4(Ph)), 7.27 (dd, 4H, $^{3}J_{obs}$ = 8.5 Hz, ^{3}J = 7.3 Hz, H3, H3'(Ph)); ^{13}C NMR ($CDCl_3$) δ 24.6 (1C, NCC**CH₂**), 29.2 (2C, NC**CH₂**), 43.7 (2C, CH_2N), 112.6 (4C, C2, C2'(Ph)), 117.1 (2C, C4(Ph)), 129.1 (4C, C3, C3'(Ph)), 148.3 (2C, C1(Ph)). MS (MALDI-TOF) calcd. for $C_{17}H_{23}N_2$: 255.19, found 255.21 $[M+H]^+$

***N*¹,*N*⁵-Di(biphenyl-4-yl)pentane-1,5-diamine** (**26**) was obtained from diamine **3** (1 mmol, 118 μL), iodobiphenyl (2.5 mmol, 700 mg) in the presence of CuI (19 mg) and 2-(isobutyryl)cyclohexanone (33 μL) in 2 mL DMF. Eluent hexanes/CH_2Cl_2 1:2, CH_2Cl_2. Yield 174 mg (43%), pale-yellow viscous oil. ^{1}H NMR ($CDCl_3$) δ 1.52-1.60 (m, 2H, $NCCCH_2$), 1.72 (quintet, 4H, ^{3}J = 7.3 Hz, $NCCH_2$), 3.20 (t, 4H, ^{3}J = 6.9 Hz, CH_2N), 3.85 (br.s, 2H, NH), 6.71 (d, 4H, $^{3}J_{obs}$ = 8.5 Hz, H(Ph)), 7.30 (t, 2H, ^{3}J = 7.3 Hz, H(Ph)), 7.43 (t, 4H, $^{3}J_{obs}$ = 7.6 Hz, H(Ph)), 7.49 (d, 4H, $^{3}J_{obs}$ = 8.5 Hz, H(Ph)), 7.58 (d, 4H, $^{3}J_{obs}$ = 7.7 Hz, H(Ph)); ^{13}C NMR ($CDCl_3$) δ 24.6 (1C, NCC**CH₂**), 29.2 (2C, NC**CH₂**), 43.9 (2C, CH_2N), 113.0 (4C, CH(Ph)), 126.0 (2C, CH(Ph)), 126.2 (4C, CH(Ph)), 127.9 (4C, CH(Ph)), 128.6 (4C, CH(Ph)), 130.2 (2C, C(Ph)), 141.2 (2C, C(Ph)), 147.6 (2C, NC(Ph)); HRMS (MALDI-TOF) calcd. for $C_{29}H_{31}N_2$: 407.2487, found 407.2462 $[M+H]^+$.

***N*¹,*N*⁵-Bis(4-fluorophenyl)pentane-1,5-diamine** (**27**) was obtained from diamine **3** (1 mmol, 118 μL), 4-iodofluorobenzene (2.5 mmol, 288 μL) in the presence of CuI (19 mg) and 2-(isobutyryl)cyclohexanone (33 μL) in 2 mL DMF. Eluent hexanes/CH_2Cl_2 1:2, CH_2Cl_2. Yield 155 mg (53%), pale-yellow viscous oil. ^{1}H NMR ($CDCl_3$) δ 1.47-1.55 (m, 2H, $NCCCH_2$), 1.66 (quintet, 4H, ^{3}J = 7.3 Hz, $NCCH_2$), 3.08 (t, 4H, ^{3}J = 6.8 Hz, CH_2N), 3.81 (br.s, 2H, NH); 6.56 (dd, 4H, $^{3}J_{HHobs}$ = 8.7 Hz, $^{4}J_{HF}$ = 4.3 Hz, H2, H2'(Ph)), 6.88 (dd, 4H, $^{3}J_{HHobs}$ = 8.7 Hz, $^{3}J_{HF}$ = 8.7 Hz, H3, H3'(Ph)); ^{13}C NMR ($CDCl_3$) δ 24.6 (1C, NCC**CH₂**), 29.2 (2C, NC**CH₂**), 44.8 (2C, CH_2N), 113.9 (d, 4C, $^{3}J_{CF}$ = 6.7 Hz, C2, C2'(Ph)), 115.7 (d, 4C, $^{2}J_{CF}$ = 21.9 Hz, C3, C3'(Ph)), 144.3 (2C, C1(Ph)), 155.9 (d, 2C, $^{1}J_{CF}$ = 235.2 Hz, C4(Ph)); HRMS (MALDI-TOF) calcd. for $C_{17}H_{21}F_2N_2$: 291.1673, found 291.1630 $[M+H]^+$.

***N*¹,*N*⁵-Bis(4-(trifluoromethyl)phenyl)pentane-1,5-diamine** (**28**) was obtained from diamine **3** (1 mmol, 118 μL), 4-iodo(trifluoromethyl)benzene (2.5 mmol, 367 μL) in the presence of CuI (19 mg) and 2-(isobutyryl)cyclohexanone (33 μL) in 2 mL DMF. Eluent hexanes/CH_2Cl_2 2:1-1:2. Yield 202 mg (51%), beige viscous oil. ^{1}H NMR ($CDCl_3$) δ 1.48-1.56 (m, 2H, $NCCCH_2$), 1.68 (quintet, 4H, ^{3}J = 7.3 Hz, $NCCH_2$), 3.16 (t, 4H, ^{3}J = 6.7 Hz, CH_2N), 3.95 (br.s, 2H, NH),

6.58 (d, 4H, $^{3}J_{obs}$ = 8.5 Hz, H2, H2'(Ph)), 7.40 (d, 4H, $^{3}J_{obs}$ = 8.5 Hz, H3, H3'(Ph)); ^{13}C NMR ($CDCl_3$) δ 24.5 (1C, NCC**CH_2**), 29.1 (2C, NC**CH_2**), 43.3 (2C, CH_2N), 111.7 (4C, C2, C2'(Ph)), 118.5 (q, 2C, $^{2}J_{CF}$ = 32.6 Hz, C4(Ph)), 125.0 (q, 2C, $^{1}J_{CF}$ = 268.9 Hz, CF_3), 126.6 (4C, C3, C3'(Ph)), 150.7 (2C, C1(Ph)); HRMS (MALDI-TOF) calcd. for $C_{19}H_{21}F_6N_2$: 391.1609, found 391.1573 $[M+H]^+$.

***N*^{1},*N*^{5}-Bis(4-methoxyphenyl)pentane-1,5-diamine** (**29**) was obtained from diamine **3** (1 mmol, 118 μL), 4-iodoanisole (2.5 mmol, 585 mg) in the presence of CuI (38 mg) and L-proline (46 mg) in 2 mL DMF. Eluent CH_2Cl_2/MeOH 25:1. Yield 165 mg (52%), brown viscous oil. ^{1}H NMR ($CDCl_3$) δ 1.45-1.54 (m, 2H, NCCCH_2), 1.65 (quintet, 4H, ^{3}J = 7.4 Hz, NCCH_2), 3.07 (t, 4H, ^{3}J = 7.1 Hz, CH_2N), 3.73 (s, 6H, OCH_3), 6.60-6.64 (m, 4H, H2, H2'(Ph)); 6.75-6.79 (m, 4H, H3, H3'(Ph)); ^{13}C NMR ($CDCl_3$) δ 24.6 (1C, NCC**CH_2**), 29.3 (2C, NC**CH_2**), 44.6 (2C, CH_2N), 55.6 (2C, CH_3O), 113.8 (4C, CH(Ph)), 114.7 (4C, CH(Ph)), 142.6 (2C, NC(Ph)), 151.7 (2C, OC(Ph)); HRMS (MALDI-TOF) calcd. for $C_{19}H_{27}N_2O_2$: 315.2073, found 315.2054 $[M+H]^+$.

***N*^{1},*N*^{6}-Diphenylhexane-1,6-diamine** (**30**) [7] was obtained from diamine **4** (1 mmol, 116 mg), iodobenzene (2.5 mmol, 280 μL) in the presence of CuI (19 mg) and 2-(isobutyryl)cyclohexanone (33 μL) in 2 mL DMF. Eluent CH_2Cl_2-CH_2Cl_2/MeOH 50:1. Yield 119 mg (45%), pale-beige crystalline powder, m.p. 79-81°C. ^{1}H NMR ($CDCl_3$) δ 1.45-1.50 (m, 4H, **CH_2**CCN), 1.66 (quintet, 4H, ^{3}J = 6.7 Hz, NC**CH_2**), 3.14 (t, 4H, ^{3}J = 7.1 Hz, CH_2NPh), 3.61 (br.s, 2H, NH), 6.61-6.65 (m, 4H, H2, H2'(Ph)), 6.72 (tt, 2H, ^{3}J = 7.3 Hz, ^{4}J = 0.8 Hz, H4(Ph)), 7.17-7.23 (m, 4H, H3, H3'(Ph)); ^{13}C NMR ($CDCl_3$) δ 26.9 (2C, C**CH_2**C), 29.5 (2C, C**CH_2**C), 43.8 (2C, CH_2NPh), 112.6 (4C, C2, C2'(Ph)), 117.1 (2C, C4(Ph)), 129.2 (4C, C3, C3'(Ph)), 148.4 (2C, C1(Ph)); HRMS (MALDI-TOF) calcd. for $C_{18}H_{25}N_2$: 269.2018, found 269.1983 $[M+H]^+$.

***N*^{1},*N*^{6}-Di(biphenyl-4-yl)hexane-1,6-diamine** (**31**) was obtained from diamine **4** (1 mmol, 116 mg), 4-iodobiphenyl (2.5 mmol, 700 mg) in the presence of CuI (19 mg) and 2-(isobutyryl)cyclohexanone (33 μL) in 2 mL DMF. Eluent CH_2Cl_2-CH_2Cl_2/MeOH 100:1-50:1. Yield 152 mg (36%), yellow crystalline powder, m.p. 144-146°C. ^{1}H NMR ($CDCl_3$) δ 1.47-1.53 (m, 4H, **CH_2**CCN), 1.69 (quintet, 4H, ^{3}J = 6.8 Hz, CH_2CN), 3.19 (t, 4H, ^{3}J = 7.0 Hz, CH_2NPh), 3.71 (br.s, 2H, NH), 6.68-6.72 (m, 4H, H(Ar)), 7.29 (tt, 2H, ^{3}J = 7.3 Hz, ^{4}J = 1.2 Hz, H(Ar)), 7.40-7.45 (m, 4H, H(Ar)), 7.46-7.50 (m, 4H, H(Ar)), 7.56-7.60 (m, 4H, H(Ar)); ^{13}C NMR ($CDCl_3$) δ 26.9 (2C, C**CH_2**C), 29.4 (2C, C**CH_2**C), 43.8 (2C, CH_2NPh), 112.9 (4C, CH(Ar)),

126.0 (2C, CH(Ar)), 126.2 (4C, CH(Ar)), 127.9 (4C, CH(Ar)), 128.6 (4C, CH(Ar)), 130.0 (2C, C(Ar)), 141.2 (2C, C(Ar)), 147.8 (2C, NC(Ar)); HRMS (MALDI-TOF) calcd. for $C_{30}H_{33}N_2$: 421.2644, found 421.2685 $[M+H]^+$.

***N*^{1},*N*^{6}-Bis(4-fluorophenyl)hexane-1,6-diamine** (**32**) was obtained from diamine **4** (1 mmol, 116 mg), 4-fluoroiodobenzene (2.5 mmol, 288 μL) in the presence of CuI (19 mg) and 2-(isobutyryl)cyclohexanone (33 μL) in 2 mL DMF. Eluent CH_2Cl_2-CH_2Cl_2/MeOH 100:1. Yield 114 mg (38%), beige crystalline powder, m.p. 94-96°C. ^{1}H NMR ($CDCl_3$) δ 1.42-1.48 (m, 4H, $\mathbf{CH_2}$CCN), 1.62 (quintet, 4H, ^3J = 6.8 Hz, CH_2CN), 3.06 (t, 4H, ^3J = 7.1 Hz, CH_2NPh), 3.48 (br.s, 2H, NH), 6.50-6.55 (m, 4H, $^4J_{HF}$ = 4.4 Hz, H2, H2'(Ph)), 6.85-6.91 (m, 4H, $^3J_{HF}$ = 8.8 Hz, H3, H3'(Ph)); ^{13}C NMR ($CDCl_3$) δ 26.9 (2C, C$\mathbf{CH_2}$C), 29.4 (2C, C$\mathbf{CH_2}$C), 44.5 (2C, CH_2NPh), 113.4 (d, 4C, $^3J_{CF}$ = 7.6 Hz, C2, C2'(Ph)), 115.6 (d, 4C, $^2J_{CF}$ = 21.9 Hz, C3, C3'(Ph)), 144.8 (2C, C1(Ph)), 155.6 (d, 2C, $^1J_{CF}$ = 235.2 Hz, C4(Ph)); HRMS (MALDI-TOF) calcd. for $C_{18}H_{23}F_2N_2$: 305.1829, found 305.1808 $[M+H]^+$.

***N*^{1}-(4-Fluorophenyl)hexane-1,6-diamine** (**33**) was obtained as the second product in the synthesis of compound **32**. Eluent CH_2Cl_2-CH_2Cl_2/MeOH 3:1 - CH_2Cl_2/MeOH/NH_3(aq) 100:20:3. Yield 121 mg (58%), brown viscous oil. ^{1}H NMR ($CDCl_3$) δ 1.36-1.41 (m, 4H, $\mathbf{CH_2}$CCN), 1.59 (quintet, 2H, ^3J = 6.8 Hz, $\mathbf{CH_2}$CNH_2), 1.74 (quintet, 2H, ^3J = 7.1 Hz, CH_2CNPh), 2.98-3.02 (m, 2H, $\mathbf{CH_2}NH_2$), 3.03 (t, 2H, ^3J = 7.1 Hz, CH_2NPh), 6.61-6.65 (m, 2H, $^4J_{HF}$ = 4.5 Hz, H2, H2'(Ph)), 6.83-6.87 (m, 2H, $^3J_{HF}$ = 8.7 Hz, H3, H3'(Ph)), NH and NH_2 protons were not unambiguously assigned; ^{13}C NMR ($CDCl_3$) δ 26.0 (1C, C$\mathbf{CH_2}$C), 26.1 (1C, C$\mathbf{CH_2}$C), 26.8 (1C, C$\mathbf{CH_2}$C), 28.4 (1C, CH_2CNPh), 40.4 (1C, CH_2NH_2), 45.4 (1C, CH_2NPh), 115.3 (d, 2C, $^3J_{CF}$ = 6.8 Hz, C2, C2'(Ph)), 115.8 (d, 2C, $^2J_{CF}$ = 21.9 Hz, C3, C3'(Ph)), 143.1 (1C, C1(Ph)), 156.6 (d, 1C, $^1J_{CF}$ = 236.9 Hz, C4(Ph)); MS (MALDI-TOF) calcd. for $C_{12}H_{20}FN_2$: 211.16, found 211.14 $[M+H]^+$.

***N*^{1},*N*^{6}-Bis(4-(trifluoromethyl)phenyl)hexane-1,6-diamine** (**34**) was obtained from diamine **4** (1 mmol, 116 mg), 1-iodo-4-(trifluoromethyl)benzene (2.5 mmol, 367 μL) in the presence of CuI (19 mg) and 2-(isobutyryl)cyclohexanone (33 μL) in 2 mL DMF. Eluent hexanes/CH_2Cl_2 4:1-1:1. Yield 139 mg (34%), beige crystalline powder, m.p. 101-103°C. ^{1}H NMR ($CDCl_3$) δ 1.42-1.49 (m, 4H, $\mathbf{CH_2}$CCN), 1.65 (quintet, 4H, ^3J = 6.8 Hz, CH_2CN), 3.15 (q, 4H, ^3J = 6.1 Hz, CH_2NPh), 3.94 (br.s, 2H, NH), 6.58 (d, 4H, $^3J_{obs}$ = 8.6 Hz, H2, H2'(Ph)), 7.39 (d, 4H, $^3J_{obs}$ = 8.6 Hz, H3, H3'(Ph)); ^{13}C NMR ($CDCl_3$) δ 26.8 (2C, $\mathbf{CH_2}$CCN), 29.2 (2C, $\mathbf{CH_2}$CN), 43.3 (2C,

CH_2NPh), 111.6 (4C, C2, C2'(Ph)), 118.5 (q, 2C, ^{2}J = 32.9 Hz, C4(Ph)), 125.1 (q, 2C, $^{1}J_{CF}$ = 270.6 Hz, CF_3), 126.6 (4C, C3, C3'(Ph)), 150.8 (2C, C1(Ph)); MS (MALDI-TOF) calcd. for $C_{20}H_{23}F_6N_2$: 405.18, found 405.17 $[M+H]^+$; HRMS (MALDI-TOF) calcd. for $C_{20}H_{22}F_5N_2$: 385.1703, found 385.1688 $[M-F]^+$.

***N*[1]-(4-(Trifluoromethyl)phenyl)hexane-1,6-diamine** (**35**) was obtained as the second product in the synthesis of compound **34**. Eluent CH_2Cl_2-CH_2Cl_2/MeOH 3:1. Yield 31 mg (12%), yellow viscous oil. ^{1}H NMR ($CDCl_3$) δ 1.28-1.36 (m, 4H, **CH_2**CCN), 1.45-1.52 (m, 2H, **CH_2**CNH_2), 1.62-1.68 (m, 2H, CH_2CNPh), 2.94 (br.s, 2H, **CH_2**NH_2), 3.13 (t, 2H, ^{3}J = 6.8 Hz, CH_2NPh), 6.65 (d, 2H, $^{3}J_{obs}$ = 8.6 Hz, H2, H2'(Ph)), 7.30 (d, 2H, $^{3}J_{obs}$ = 8.6 Hz, H3, H3'(Ph)), NH and NH_2 protons were not unambiguously assigned; ^{13}C NMR ($CDCl_3$) δ 27.2 (1C, CH_2), 27.6 (1C, CH_2), 28.5 (1C, CH_2), 29.7 (1C, CH_2), 40.7 (1C, CH_2NH_2), 43.8 (1C, CH_2Ph), 112.5 (2C, C2, C2'(Ph)), 127.2 (2C, C3, C3'(Ph)), 153.1 (1C, C1(Ph)), quaternary carbon atoms C4(Ph) and CF_3 were not unambiguously assigned due to very low intensity of corresponding quadruplets. MS (MALDI-TOF) calcd. for $C_{13}H_{20}F_3N_2$: 261.16, found 261.18 $[M+H]^+$. HRMS (MALDI-TOF) calcd. for $C_{13}H_{19}F_2N_2$: 241.1516, found 241.1537 $[M-F]^+$.

***N*[1],*N*[6]-Bis(4-methoxyphenyl)hexane-1,6-diamine** (**36**) was obtained from diamine **4** (1 mmol, 116 mg), 4-iodoanisole (2.5 mmol, 585 mg) in the presence of CuI (38 mg) and 2-(isobutyryl)cyclohexanone (67 μL) in 2 mL DMF. Eluent hexanes/CH_2Cl_2 2:1 – CH_2Cl_2/MeOH 50:1. Yield 265 mg (81%), brown crystalline powder, m.p. 96-98°C. ^{1}H NMR ($CDCl_3$) δ 1.41-1.47 (m, 4H, **CH_2**CCN), 1.62 (quintet, 4H, ^{3}J = 6.6 Hz, **CH_2**CN), 3.06 (t, 4H, ^{3}J = 7.1 Hz, CH_2NPh), 3.74 (s, 6H, CH_3), 6.55-6.59 (m, 4H, H2, H2'(Ph)), 6.75-6.79 (m, 4H, H3, H3'(Ph)), NH protons were not unambiguously assigned; ^{13}C NMR ($CDCl_3$) δ 26.9 (2C, **CH_2**CCN), 29.5 (2C, CH_2CN), 44.8 (2C, CH_2NPh), 55.7 (2C, OCH_3), 113.9 (4C, CH(Ph)), 114.8 (4C, CH(Ph)), 142.7 (2C, C1(Ph)), 151.9 (2C, C4(Ph)); HRMS (MALDI-TOF) calcd. for $C_{20}H_{29}N_2O_2$: 329.2229, found 329.2256 $[M+H]^+$.

***N*[1]-(2-Fluorophenyl)propane-1,3-diamine** (**37**) [8] was obtained from diamine **1** (1 mmol, 83 μL), 2-fluoroiodobenzene (2.5 mmol, 292 μL) in the presence of CuI (38 mg) and 2-(isobutyryl)cyclohexanone (67 μL) in 2 mL DMF. Eluent CH_2Cl_2/MeOH 3:1. Yield 99 mg (58%), pale-yellow viscous oil. ^{1}H NMR ($CDCl_3$) δ 1.85 (quintet, 2H, ^{3}J = 6.5 Hz, C**CH_2**C), 2.87 (br.s, 2H, **CH_2**NH_2), 3.17 (br.s, 2H, CH_2NPh), 5.28 (br.s, 1H, NH), 6.45-6.52 (m, 1H, H4(Ph)), 6.67 (dd, 1H, ^{3}J = 8.5 Hz, $^{4}J_{HF}$ = 8.5 Hz, H6(Ph)), 6.86-6.93 (m, 2H, H3, H5(Ph)), NH_2

protons were not unambiguously assigned; ^{13}C NMR ($CDCl_3$) δ 26.2 (1C, C**CH_2**C), 36.8 (1C, CH_2NH_2), 39.2 (1C, CH_2NPh), 111.5 (1C, CH(Ph)), 114.0 (d, 1C, $^2J_{CF}$ = 18.5 Hz, C3(Ph)), 115.2 (d, 1C, $^3J_{CF}$ = 5.9 Hz, CH(Ph)), 124.4 (1C, C5(Ph)), 136.2 (d, 1C, $^2J_{CF}$ = 11.0 Hz, C1(Ph)), 150.8 (d, 1C, $^1J_{CF}$ = 237.7 Hz, C2(Ph)).

***N*^{1},*N*^{5}-Bis(2-fluorophenyl)pentane-1,4-diamine** (**38**) was obtained from diamine **3** (1 mmol, 118 μL), 2-fluoroiodobenzene (2.5 mmol, 292 μL) in the presence of CuI (38 mg) and 2-(isobutyryl)cyclohexanone (67 μL) in 2 mL DMF. Eluent hexanes/CH_2Cl_2 1:1. Yield 30 mg (10%), pale-yellow crystalline powder, m.p. 54-56°C. ^{1}H NMR ($CDCl_3$) δ 1.49-1.58 (m, 2H, **CH_2**CCN), 1.70 (quintet, 4H, ^3J = 7.3 Hz, CH_2CN), 3.16 (t, 4H, ^3J = 7.0 Hz, CH_2NPh), 3.87 (br.s, 2H, NH), 6.60 (tdd, 2H, ^3J = 8.1 Hz, ^4J = 1.7 Hz, $^4J_{HF}$ = 4.9 Hz, H4(Ph)), 6.65-6.71 (m, 2H, ^4J = 1.7 Hz, H6(Ph)), 6.96 (ddd, 2H, ^3J = 8.1 Hz, ^4J = 1.4 Hz, $^3J_{HF}$ = 12.0 Hz, H3(Ph)), 6.99 (tdd, 2H, ^3J = 8.1 Hz, ^4J = 1.4 Hz, $^5J_{HF}$ = 0.6 Hz, H5(Ph)); ^{13}C NMR ($CDCl_3$) δ 24.6 (1C, **CH_2**CCN), 29.2 (2C, **CH_2**CN), 43.4 (2C, CH_2NPh), 111.9 (2C, CH(Ph)), 114.3 (d, 2C, $^2J_{CF}$ = 18.6 Hz, C3(Ph)), 116.3 (d, 2C, $^3J_{CF}$ = 5.9 Hz, CH(Ph)), 124.5 (2C, C5(Ph)), 136.8 (d, 2C, $^2J_{CF}$ = 11.0 Hz, C1(Ph)), 151.4 (d, 2C, $^1J_{CF}$ = 237.2 Hz, C2(Ph)); HRMS (MALDI-TOF) calcd. for $C_{17}H_{21}F_2N_2$: 291.1673, found 291.1645 $[M+H]^+$.

***N*^{1}-(2-Fluorophenyl)pentane-1,4-diamine** (**39**) was obtained as the main product in the synthesis of compound **38**. Eluent CH_2Cl_2/MeOH 25:1-3:1. Yield 74 mg (38%), pale-yellow viscous oil. ^{1}H NMR ($CDCl_3$) δ 1.39-1.47 (m, 2H, CH_2), 1.51-1.59 (m, 2H, CH_2), 1.61-1.69 (m, 2H, CH_2), 3.09-3.16 (m, 4H, CH_2N), 3.90 (br.s, 1H, NH), 6.55-6.61 (m, 1H, H4(Ph)), 6.63-6.68 (m, 1H, H6(Ph)), 6.90-6.99 (m, 2H, H3, H5(Ph)), NH_2 protons were not unambiguously assigned; ^{13}C NMR ($CDCl_3$) δ 24.3 (1C, CH_2), 29.0 (1C, CH_2), 29.3 (1C, CH_2), 43.3 (2C, CH_2N), 111.9 (1C, CH(Ph)), 114.3 (d, 1C, $^2J_{CF}$ = 18.6 Hz, C3(Ph)), 116.3 (d, 1C, $^3J_{CF}$ = 6.8 Hz, CH(Ph)); 124.5 (1C, C5(Ph)); 138.6 (br.s, 1C, C1(Ph)), 151.5 (d, 1C, $^1J_{CF}$ = 238.6 Hz, C2(Ph)); MS (MALDI-TOF) calcd. for $C_{11}H_{18}FN_2$ 197.15, found 197.14 $[M+H]^+$.

***N*^{1},*N*^{6}-Bis(2-fluorophenyl)hexane-1,6-diamine** (**40**) was obtained from diamine **4** (1 mmol, 116 mg), 2-fluoroiodobenzene (2.5 mmol, 292 μL) in the presence of CuI (38 mg) and 2-(isobutyryl)cyclohexanone (67 μL) in 2 mL DMF. Eluent hexanes/CH_2Cl_2 1:1-1:2. Yield 56 mg (18%), white crystalline powder, m.p. 55-57°C. ^{1}H NMR ($CDCl_3$) δ 1.45-1.50 (m, 4H, **CH_2**CCN), 1.67 (quintet, 4H, ^3J = 6.7 Hz, CH_2CN), 3.14 (t, 4H, ^3J = 7.0 Hz, CH_2NPh), 3.84 (br.s, 2H, NH), 6.56-6.63 (m, 2H, H4(Ph)), 6.68 (dd, 2H, $^3J_{obs}$ = 8.3 Hz, $^4J_{HF}$ = 8.3 Hz, H6(Ph)),

6.95 (dd, 2H, $^{3}J_{obs}$ = 8.0 Hz, $^{3}J_{HF}$ = 12.1 Hz, H3(Ph)), 6.98 (t, 2H, $^{3}J_{obs}$ = 7.7 Hz, H5(Ph)); ^{13}C NMR ($CDCl_3$) δ 26.9 (2C, **CH_2**CCN), 29.4 (2C, **CH_2**CN), 43.5 (2C, CH_2NPh), 112.0 (2C, CH(Ph)), 114.3 (d, 2C, $^{2}J_{CF}$ = 18.6 Hz, H3(Ph)), 116.3 (d, 2C, $^{3}J_{CF}$ = 6.7 Hz, CH(Ph)), 124.6 (2C, C5(Ph)), 137.0 (d, 2C, $^{2}J_{CF}$ = 11.8 Hz, C1(Ph)), 151.5 (d, 2C, $^{1}J_{CF}$ = 237.7 Hz, C2(Ph)); HRMS (MALDI-TOF) calcd. for $C_{18}H_{23}F_2N_2$ 305.1829, found 305.1838 $[M+H]^+$.

***N*^{1}-(2-Fluorophenyl)hexane-1,6-diamine** (**41**) was obtained as the main product in the synthesis of compound **40**. Eluent CH_2Cl_2/MeOH 5:1-3:1. Yield 66 mg (31%), pale-yellow viscous oil. ^{1}H NMR ($CDCl_3$) δ 1.38-1.46 (m, 4H, **CH_2**CCN), 1.62 (quintet, 2H, ^{3}J = 6.9 Hz, **CH_2**CNH_2), 1.87 (quintet, 2H, ^{3}J = 7.2 Hz, CH_2CNPh), 3.07-3.15 (m, 4H, CH_2N), 3.68 (br.s, 1H, NHPh), 6.55-6.61 (m, 1H, H4(Ph)), 6.67 (ddd, 1H, $^{3}J_{obs}$ = 8.4 Hz, ^{4}J = 1.5 Hz, $^{4}J_{HF}$ = 8.4 Hz, H6(Ph)), 6.93 (ddd, 1H, ^{3}J = 8.0 Hz, ^{4}J = 1.4 Hz, $^{3}J_{HF}$ = 11.0 Hz, H3(Ph)), 6.96 (t, 1H, $^{3}J_{obs}$ = 7.6 Hz, H5(Ph)), NH_2 protons were not unambiguously assigned; ^{13}C NMR ($CDCl_3$) δ 27.2 (1C, CH_2), 27.5 (1C, CH_2), 28.4 (1C, CH_2), 30.0 (1C, CH_2), 40.7 (1C, CH_2NH_2), 44.3 (1C, CH_2NPh), 113.5 (1C, CH(Ph)), 115.1 (d, 1C,$^{2}J_{CF}$ = 19.4 Hz, C3(Ph)), 117.1 (d, 1C, $^{3}J_{CF}$ = 5.9 Hz, CH(Ph)), 125.6 (1C, C5(Ph)), 138.2 (d, 1C, $^{2}J_{CF}$ = 11.8 Hz, C1(Ph)), 153.0 (d, 1C, $^{1}J_{CF}$ = 237.7 Hz, C2(Ph)); HRMS (MALDI-TOF) calcd. for $C_{12}H_{20}FN_2$ 211.1611, found 211.1569 $[M+H]^+$.

***N*^{1}-Phenyl-*N*^{3}-(3-(phenylamino)propyl)propane-1,3-diamine** (**42**) was obtained in two reactions: a) from triamine **5** (0.5 mmol, 72 μL), iodobenzene (1.25 mmol, 140 μL) in the presence of CuI (9.5 mg) and L-proline (11.5 mg) in 1 mL EtCN; b) from triamine **5** (0.5 mmol, 72 μL), iodobenzene (1.25 mmol, 140 μL) in the presence of CuI (9.5 mg) and L-proline (11.5 mg) in 1 mL DMF. Chromatographic isolation of combined reaction mixtures: eluent CH_2Cl_2/MeOH 3:1 - CH_2Cl_2/MeOH/NH_3(aq) 100:20:1. Yield 116 mg (41%), pale-yellow viscous oil. ^{1}H NMR ($CDCl_3$) δ 1.90 (quintet, 4H, ^{3}J = 6.6 Hz, C**CH_2**C), 2.84 (t, 4H, ^{3}J = 6.6 Hz, CH_2NCH_2), 3.21 (t, 4H, ^{3}J = 6.6 Hz, CH_2NPh), 4.09 (br.s, 2H, NHPh), 6.61 (d, 4H, $^{3}J_{obs}$ = 8.5 Hz, H2, H2'(Ph)), 6.71 (t, 2H, ^{3}J = 7.3 Hz, H4(Ph)), 7.17 (dd, 4H, $^{3}J_{obs}$ = 8.5 Hz, $^{3}J_{obs}$ = 7.3 Hz, H3, H3'(Ph)), NH proton of the dialkylamino group was not unambiguously assigned; ^{13}C NMR ($CDCl_3$) δ 28.2 (2C, C**CH_2**C), 42.2 (2C, CH_2NPh), 47.8 (2C, CH_2NCH_2), 112.8 (4C, C2, C2'(Ph)), 117.3 (2C, C4(Ph)), 129.2 (4C, C3, C3'(Ph)), 148.2 (2C, C1(Ph)); HRMS (MALDI-TOF) calcd. for $C_{18}H_{26}N_3$ 284.2127, found 284.2093 $[M+H]^+$.

***N*^{1}-(Biphenyl-4-yl)-*N*^{3}-(3-(biphenyl-4-ylamino)propyl)propane-1,3-diamine** (**43**) was obtained from triamine **5** (1 mmol, 143 μL), 4-iodobiphenyl (2.5 mmol, 700 mg) in the presence of CuI (38 mg) and *L*-proline (46 mg) in 2 mL EtCN. Eluent CH_2Cl_2/MeOH 3:1. Yield 326 mg

(75%), pale-yellow viscous oil. ^{1}H NMR ($CDCl_3$) δ 1.84 (quintet, 4H, ^3J = 6.6 Hz, C**CH$_2$**C), 2.79 (t, 4H, ^3J = 6.6 Hz, CH_2NCH_2), 3.26 (t, 4H, ^3J = 6.6 Hz, CH_2NPh), 4.28 (br.s, 2H, NHPh); 6.65-6.69 (m, 4H, H(Ph)), 7.24 (tt, 2H, ^3J = 7.3 Hz, ^4J = 1.2 Hz, H(Ph)), 7.35-7.40 (m, 4H, H(Ph)), 7.41-7.45 (m, 4H, H(Ph)), 7.50-7.54 (m, 4H, H(Ph)), NH proton of the dialkylamino group was not unambiguously assigned; ^{13}C NMR ($CDCl_3$) δ 29.6 (2C, C**CH$_2$**C), 42.9 (2C, CH_2NPh), 48.4 (2C, CH_2NCH_2), 113.0 (4C, CH(Ph)), 126.0 (2C, CH(Ph)), 126.2 (4C, CH(Ph)), 127.9 (4C, CH(Ph)), 128.6 (4C, CH(Ph)), 130.0 (2C, C(Ph)), 141.3 (2C, C(Ph)), 147.9 (2C, NC(Ph)); HRMS (MALDI-TOF) calcd. for $C_{30}H_{34}N_3$ 436.2753, found 436.2776 $[M+H]^+$.

***N*^{1}-(3-Aminopropyl)-*N*^{3}-(biphenyl-4-yl)propane-1,3-diamine** (**44**) was obtained as the second product in the synthesis of compound **43** in the presence of CuI (19 mg) and L-proline (23 mg) in 2 mL EtCN. Eluent CH_2Cl_2/MeOH/NH_3(aq) 10:4:1. Yield 51 mg (18%), brown viscous oil. ^{1}H NMR ($CDCl_3$) δ 1.74 (quintet, 2H, ^3J = 5.8 Hz, **CH$_2$**CH_2NH_2), 1.87 (quintet, 2H, ^3J = 6.4 Hz, **CH$_2$**CH_2NHPh), .2.75-2.89 (m, 6H, CH_2N), 3.22 (t, 2H, ^3J = 6.4 Hz, CH_2NHPh), 3.58 (br.s, 4H, NH, NH_2), 6.67 (d, 2H, $^3J_{obs}$ = 8.5 Hz, H(Ph)), 7.22 (t, 1H, ^3J = 7.3 Hz, H(Ph)), 7.36 (t, 2H, $^3J_{obs}$ = 7.6 Hz, H(Ph)), 7.41 (d, 2H, $^3J_{obs}$ = 8.5 Hz, H(Ph)), 7.50 (d, 2H, $^3J_{obs}$ = 8.1 Hz, H(Ph)); ^{13}C NMR ($CDCl_3$) δ 28.9 (1C, **CH$_2$**CH_2NPh), 32.3 (1C, **CH$_2$**CH_2NH_2), 40.6 (1C, CH_2NH_2), 42.7 (1C, CH_2NPh), 48.2 (2C, CH_2NHCH_2), 112.9 (2C, CH(Ph)), 126.0 (1C, CH(Ph)), 126.2 (2C, CH(Ph)), 127.9 (2C, CH(Ph)), 128.6 (2C, CH(Ph)), 129.9 (1C, C(Ph)), 141.2 (1C, C(Ph)), 147.9 (1C, NC(Ph)); HRMS (MALDI-TOF) calcd. for $C_{18}H_{26}N_3$ 284.2122, found 284.2157 $[M+H]^+$.

***N*^{1}-(4-Fluorophenyl)-*N*^{3}-(3-(4-fluorophenylamino)propyl)propane-1,3-diamine** (**45**) was obtained in two reactions: a) from triamine **5** (0.5 mmol, 72 μL), 4-fluoroiodobenzene (1.25 mmol, 144 μL) in the presence of CuI (9.5 mg) and L-proline (11.5 mg) in 1 mL EtCN; b) from triamine **5** (0.5 mmol, 72 μL), 4-fluoroiodobenzene (1.25 mmol, 144 μL) in the presence of CuI (19 mg) and L-proline (23 mg) in 1 mL EtCN. Chromatographic isolation of combined reaction mixtures: eluent CH_2Cl_2/MeOH 3:1 - CH_2Cl_2/MeOH/NH_3(aq) 100:20:2. Yield 164 mg (50%), pale-yellow viscous oil. ^{1}H NMR ($CDCl_3$) δ 1.92 (quintet, 4H, ^3J = 6.6 Hz, C**CH$_2$**C), 2.87 (t, 4H, ^3J = 6.6 Hz, CH_2NCH_2), 3.17 (t, 4H, ^3J = 6.6 Hz, CH_2NPh), 4.35 (br.s, 2H, NHPh), 6.52 (dd, 4H, $^3J_{obs}$ = 8.7 Hz, $^4J_{HF}$ = 4.4 Hz, H2, H2'(Ph)), 6.85 (dd, 4H, $^3J_{obs}$ = $^3J_{HF}$ = 8.7 Hz, H3, H3'(Ph)), NH proton of the dialkylamino group was not unambiguously assigned; ^{13}C NMR ($CDCl_3$) δ 27.9 (2C, C**CH$_2$**C), 42.9 (2C, CH_2NPh), 47.7 (2C, CH_2NCH_2), 113.6 (d, 4C, $^3J_{CF}$ = 6.8 Hz, C2, C2'(Ph)), 115.7 (d, 4C, $^2J_{CF}$ = 22.8 Hz, C3, C3'(Ph)), 144.5 (2C, C1(Ph)), 155.7 (d, 2C, $^1J_{CF}$ =

234.4 Hz, C4(Ph)); HRMS (MALDI-TOF) calcd. for $C_{18}H_{24}F_2N_3$ 320.1938, found 320.1915 $[M+H]^+$.

***N*^{1}-(4-(Trifluoromethyl)phenyl)-*N*^{3}-(3-(4-(trifluoromethyl)phenylamino)propyl)propane-1,3-diamine** (**46**) was obtained from triamine **5** (1 mmol, 143 μL), 4-iodo(trifluoromethyl)benzene (2.5 mmol, 367 μL) in the presence of CuI (19 mg) and L-proline (23 mg) in 2 mL EtCN. Eluent CH_2Cl_2/MeOH 5:1 - CH_2Cl_2/MeOH/NH_3(aq) 100:20:1. Yield 225 mg (53%), pale-yellow viscous oil. ^{1}H NMR ($CDCl_3$) δ 1.81 (quintet, 4H, ^3J = 6.5 Hz, C**CH$_2$**C), 2.76 (t, 4H, ^3J = 6.5 Hz, CH_2NCH_2), 3.23 (t, 4H, ^3J = 6.5 Hz, CH_2NPh), 4.59 (br.s, 2H, NHPh); 6.56 (d, 4H, $^3J_{obs}$ = 8.6 Hz, H2, H2'(Ph)), 7.37 (d, 4H, $^3J_{obs}$ = 8.6 Hz, H3, H3'(Ph)), NH proton of the dialkylamino group was not unambiguously assigned; ^{13}C NMR ($CDCl_3$) δ 29.2 (2C, C**CH$_2$**C), 42.4 (2C, CH_2NPh), 48.2 (2C, CH_2NCH_2), 111.7 (4C, C2, C2'(Ph)), 118.5 (q, 2C, $^2J_{CF}$ = 32.2 Hz, C4(Ph)), 125.1 (2C, $^1J_{CF}$ = 281.0 Hz, CF_3), 126.6 (4C, C3, C3'(Ph)), 150.9 (2C, C1(Ph)); HRMS (MALDI-TOF) calcd. for $C_{20}H_{24}F_6N_3$ 420.1874, found 420.1898 $[M+H]^+$. By the treatment with 5M HCl solution in aqueous dioxane-methanol solution corresponding hydrochloride as dihydrate was obtained as white crystalline powder, m.p. 178-180°C. Calcd. for $C_{20}H_{24}ClF_6N_3*2H_2O$ (%): C 48.83, H 5.74, N 8.54; found C 48.69, H 6.20, N 8.80.

***N*^{1}-(4-Methoxyphenyl)-*N*^{3}-(3-(4-methoxyphenylamino)propyl)propane-1,3-diamine** (**47**) was obtained from triamine **5** (1 mmol, 143 μL), 4-iodoanisole (2.5 mmol, 585 mg) in the presence of CuI (38 mg) and L-proline (46 mg) in 2 mL EtCN. Eluent CH_2Cl_2/MeOH 5:1 – 3:1. Yield 73 mg (21%), pale-beige crystalline powder, m.p. 195-197°C. ^{1}H NMR (DMSO-d_6) δ 1.86 (quintet, 4H, ^3J = 7.1 Hz, C**CH$_2$**C), 2.97 (t, 4H, ^3J = 7.5 Hz, **CH$_2$**NH**CH$_2$**), 3.04 (t, 4H, ^3J = 6.6 Hz, CH_2NPh), 3.62 (s, 6H, OCH_3), 6.50-6.54 (m, 4H, H2, H2'(Ph)), 6.67-6.71 (m, 4H, H3, H3'(Ph)), NH protons were not unambiguously assigned; ^{13}C NMR (DMSO-d_6) δ 25.2 (2C, C**CH$_2$**C), 40.7 (2C, CH_2NPh), 45.1 (2C, **CH$_2$**NH**CH$_2$**), 55.2 (2C, OCH_3), 113.1 (4C, CH(Ph)), 114.5 (4C, CH(Ph)), 142.7 (2C, C1(Ph)), 150.8 (2C, C4(Ph)); HRMS (MALDI-TOF) calcd. for $C_{20}H_{30}N_3O_2$ 344.2338, found 344.2307 $[M+H]^+$.

***N*^{1}-(3-Aminopropyl)-*N*^{3}-(4-methoxyphenyl)propane-1,3-diamine** (**48**) was obtained as the second product in the synthesis of compound **47**. Eluent CH_2Cl_2/MeOH/NH_3(aq) 100:20:1 – 100:20:2, 10:4:1. Yield 82 mg (35%), yellow viscous oil. ^{1}H NMR (DMSO-d_6) δ 1.81 (quintet, 2H, ^3J = 6.9 Hz, **CH$_2$**CNH$_2$), 1.87 (quintet, 2H, ^3J = 6.9 Hz, CH_2CNPh), 2.80-2.87 (m, 4H, **CH$_2$**NH**CH$_2$**), 2.88 (t, 2H, ^3J = 7.1 Hz, **CH$_2$**NH$_2$), 3.02 (t, 2H, ^3J = 6.6 Hz, CH_2NPh), 3.62 (s, 3H, OCH_3), 6.48-6.52 (m, 2H, H2, H2'(Ph)), 6.65-6.69 (m, 2H, H3, H3'(Ph)), NH and NH_2

protons were not unambiguously assigned; ^{13}C NMR (DMSO-d_6) δ 24.9 (1C, **CH$_2$**CNPh), 26.5 (1C, **CH$_2$**CNH$_2$), 36.9 (1C, CH$_2$NH$_2$), 41.2 (1C, CH$_2$NPh), 44.9 (1C, **CH$_2$**NHCH$_2$), 45.8 (1C, CH$_2$NH**CH$_2$**), 55.2 (1C, OCH$_3$), 113.0 (2C, CH(Ph)), 114.4 (2C, CH(Ph)), 142.9 (1C, C1(Ph)), 150.6 (1C, C4(Ph)); MS (MALDI-TOF) calcd. for $C_{13}H_{24}N_3O$ 238.19, found 238.21 [M+H]$^+$.

***N*^{1}-Phenyl-*N*^{4}-(3-(phenylamino)propyl)butane-1,4-diamine** (**49**) was obtained from triamine **6** (1 mmol, 152 μL), iodobenzene (2.5 mmol, 280 μL) in the presence of CuI (19 mg) and L-proline (23 mg) in 2 mL EtCN. Eluent CH_2Cl_2/MeOH 3:1 - CH_2Cl_2/MeOH/NH_3(aq) 10:4:1. Yield 108 mg (36%), pale-yellow viscous oil. ^{1}H NMR (CDCl$_3$) δ 1.63-1.70 (m, 4H, CCH$_2$CH$_2$C), 1.86 (quintet, 2H, ^3J = 6.6 Hz, C**CH$_2$**C), 2.67-2.73 (m, 2H, CH$_2$N), 2.80 (t, 2H, ^3J = 6.7 Hz, CH$_2$N), 3.08-3.13 (m, 2H, CH$_2$NPh), 3.20 (t, 2H, ^3J = 6.5 Hz, CH$_2$NPh), 3.55 (br.s, 2H, NHPh), 6.57-6.62 (m, 4H, H2, H2'(Ph)), 6.69 (t, 2H, ^3J = 7.3 Hz, H4(Ph)), 7.13-7.19 (m, 4H, H3, H3'(Ph)), NH proton of the dialkylamino group was not unambiguously assigned; ^{13}C NMR (CDCl$_3$) δ 27.0 (1C, C**CH$_2$**CH$_2$C), 27.1 (1C, CCH$_2$**CH$_2$**C), 28.7 (1C, C**CH$_2$**C), 42.5 (1C, CH$_2$NPh), 43.6 (1C, CH$_2$NPh); 48.0 (1C, CH$_2$N), 49.3 (1C, CH$_2$N), 112.7 (4C, C2, C2'(Ph)), 117.2 (2C, C4(Ph)), 129.2 (4C, C3, C3'(Ph)), 148.4 (2C, C1(Ph)); HRMS (MALDI-TOF) calcd. for $C_{19}H_{28}N_3$ 298.2283, found 298.2315 [M+H]$^+$. By the treatment with 5M HCl solution in aqueous dioxane-methanol solution corresponding trihydrochloride as hydrate was obtained as white crystalline powder, m.p. 210°C (decomp.). Calcd. for $C_{19}H_{30}Cl_3N_3{*}H_2O$ (%):C 53.72, H 7.59, N 9.89; found C 54.40, H 7.46, N 9.86.

***N*^{1}-(Biphenyl-4-yl)-*N*^{4}-(3-(biphenyl-4-ylamino)propyl)butane-1,4-diamine** (**50**) was obtained from triamine **6** (1 mmol, 152 μL), 4-iodobiphenyl (2.5 mmol, 700 mg) in the presence of CuI (38 mg) and L-proline (46 mg) in 2 mL EtCN. Eluent CH_2Cl_2/MeOH/NH_3(aq) 100:20:1. Yield 157 mg (35%), yellow viscous oil. ^{1}H NMR (CDCl$_3$) δ 1.60-1.75 (m, 4H, CCH$_2$CH$_2$C), 1.84 (quintet, 2H, ^3J = 6.6 Hz, C**CH$_2$**C), 2.69 (t, 2H, ^3J = 6.8 Hz, CH$_2$N), 2.79 (t, 2H, ^3J = 6.6 Hz, CH$_2$N), 3.18 (t, 2H, ^3J = 6.7 Hz, CH$_2$NPh), 3.25 (t, 2H, ^3J = 6.5 Hz, CH$_2$N), 6.64-6.70 (m, 4H, H(Ar)), 7.24 (t, 2H, ^3J = 7.3 Hz, H(Ar)), 7.35-7.41 (m, 4H, H(Ar)), 7.42-7.45 (m, 4H, H(Ar)); 7.51-7.55 (m, 4H, H(Ar)), NH protons were not unambiguously assigned; ^{13}C NMR (CDCl$_3$) δ 27.2 (1C, CCH$_2$CH$_2$C), 27.5 (1C, CCH$_2$CH$_2$C), 29.2 (1C, CCH$_2$C), 43.7 (2C, CH$_2$NPh), 48.3 (1C, CH$_2$N), 49.6 (1C, CH$_2$N), 112.9 (4C, CH(Ar)), 125.9 (2C, CH(Ar)), 126.2 (4C, CH(Ar)), 127.9 (4C, CH(Ar)), 128.6 (4C, CH(Ar)), 129.9 (2C, C(Ar)), 141.2 (2C, C(Ar)), 147.8 (1C, NC(Ar)), 147.9 (1C, NC(Ar)); HRMS (MALDI-TOF) calcd. for $C_{31}H_{36}N_3$ 450.2909, found 450.2946 [M+H]$^+$.

***N*^1^-(4-Fluorophenyl)-*N*^4^-(3-(4-fluorophenylamino)propyl)butane-1,4-diamine** (**51**) was obtained from triamine **6** (1 mmol, 152 μL), 4-fluoroiodobenzene (2.5 mmol, 288 μL) in the presence of CuI (19 mg) and L-proline (23 mg) in 2 mL EtCN. Eluent CH_2Cl_2/MeOH/NH_3(aq) 100:20:3-100:25:5. Yield 118 mg (35%), pale-yellow viscous oil. ^{1}H NMR ($CDCl_3$) δ 1.58-1.69 (m, 4H, CCH_2CH_2C), 1.81 (quintet, 2H, ^3J = 6.6 Hz, C**CH₂**C), 2.67 (t, 2H, ^3J = 6.6 Hz, CH_2N), 2.77 (t, 2H, ^3J = 6.6 Hz, CH_2N), 3.07 (t, 2H, ^3J = 6.6 Hz, CH_2NPh), 3.14 (t, 2H, ^3J = 6.6 Hz, CH_2N), 3.20 (br.s, 2H, NHPh), 6.50 (dd, 2H, $^3J_{obs}$ = 9.0 Hz, $^4J_{HF}$ = 4.4 Hz, H2, H2'(Ph)), 6.51 (dd, 2H, $^3J_{obs}$ = 9.0 Hz, $^4J_{HF}$ = 4.4 Hz, H2, H2'(Ph)), 6.83-6.90 (m, 4H, H3, H3'(Ph)), NH proton of dialkylamino group was not unambiguously assigned; ^{13}C NMR ($CDCl_3$) δ 27.2 (2C, C**CH₂CH₂**C), 27.4 (1C, C**CH₂**C), 43.5 (1C, CH_2NPh), 44.4 (1C, CH_2NPh), 48.2 (1C, CH_2N), 49.5 (1C, CH_2N), 113.4 (4C, C2, C2'(Ph)), 115.6 (d, 4C, $^2J_{CF}$ = 15.2 Hz, C3, C3'(Ph)), 144.8 (1C, C1(Ph)), 144.9 (1C, C1(Ph)), 155.7 (d, 2C, $^1J_{CF}$ = 234.4 Hz, C4(Ph)); HRMS (MALDI-TOF) calcd. for $C_{19}H_{26}F_2N_3$ 334.2095, found 334.2077 $[M+H]^+$.

***N*^1^-(4-(Trifluoromethyl)phenyl)-*N*^4^-(3-(4-(trifluoromethyl)phenylamino)propyl)butane-1,4-diamine** (**52**) was obtained from triamine **6** (1 mmol, 152 μl), 4-iodo(trifluoromethyl)benzene (2.5 mmol, 367 μL) in the presence of CuI (19 mg) and L-proline (23 mg) in 2 mL EtCN. Eluent CH_2Cl_2/MeOH/NH_3(aq) 100:20:1-100:20:2. Yield 230 mg (51%), pale-yellow viscous oil. ^{1}H NMR ($CDCl_3$) δ 1.64-1.73 (m, 4H, CCH_2CH_2C), 1.88 (quintet, 2H, ^3J = 6.5 Hz, C**CH₂**C), 2.73 (t, 2H, ^3J = 6.6 Hz, CH_2N), 2.83 (t, 2H, ^3J = 6.6 Hz, CH_2N), 3.12 (t, 2H, ^3J = 6.3 Hz, CH_2NPh), 3.22 (t, 2H, ^3J = 6.5 Hz, CH_2NPh), 6.56 (d, 4H, $^3J_{obs}$ = 8.6 Hz, H2, H2'(Ph)), 7.36 (d, 4H, $^3J_{obs}$ = 8.6 Hz, H3, H3'(Ph)), NH protons were not unambiguously assigned; ^{13}C NMR ($CDCl_3$) δ 26.6 (2C, C**C**H₂**C**H₂C), 28.0 (1C, C**C**H₂C); 42.0 (1C, CH_2NPh), 42.9 (1C, CH_2NPh); 47.6 (1C, CH_2N), 49.0 (1C, CH_2N), 111.5 (4C, C2, C2'(Ph)), 118.0 (q, 2C, $^2J_{CF}$ = 32.3 Hz, C4(Ph)), 125.5 (q, 2C, $^1J_{CF}$ = 270.0 Hz, CF_3), 126.4 (4C, C3, C3'(Ph)), 150.7 (1C, C1(Ph)), 150.8 (1C, C1(Ph)); HRMS (MALDI-TOF) calcd. for $C_{21}H_{26}F_6N_3$ 434.2031, found 434.2055 $[M+H]^+$. By the treatment with 5M HCl solution in aqueous dioxane-methanol solution corresponding trihydrochloride was obtained as pale-beige crystalline powder, m.p. 162-164°C (decomp.). Calcd. for $C_{21}H_{28}Cl_3F_6N_3$ (%): C 46.47, H 5.20, N 7.74; found C 45.88, H 5.18, N 7.65.

***N*^1^-(4-Methoxyphenyl)-*N*^4^-(3-(4-methoxyphenylamino)propyl)butane-1,4-diamine** (**53**) was obtained from triamine **6** (1 mmol, 152 μL), 4-iodoanisole (2.5 mmol, 585 mg) in the presence of CuI (38 mg) and 2-(isobutyryl)cyclohexanone (67 μL) in 2 mL DMF. Eluent CH_2Cl_2/MeOH/NH_3(aq) 100:20:2-100:20:3. Yield 163 mg (46%), yellow viscous oil. ^{1}H NMR

($CDCl_3$) δ 1.60-1.68 (m, 4H, C**CH_2CH_2**C), 1.82 (quintet, 2H, ^3J = 6.6 Hz, C**CH_2**C), 2.65-2.70 (m, 2H, CH_2N); 2.77 (t, 2H, ^3J = 6.7 Hz, CH_2N); 3.03-3.09 (m, 2H, CH_2NPh), 3.13 (t, 2H, ^3J = 6.5 Hz, CH_2NPh), 3.31 (br.s, 2H, NHPh), 3.72 (s, 6H, OCH_3), 6.54-6.58 (m, 4H, H2, H2'(Ph)), 6.73-6.78 (m, 4H, H3, H3'(Ph)), NH proton of dialkylamino group was not unambiguously assigned; ^{13}C NMR ($CDCl_3$) δ 27.1 (2C, CCH_2CH_2C), 27.2 (1C, CCH_2CH_2C), 43.5 (1C, CH_2NPh), 44.7 (1C, CH_2NPh), 48.0 (1C, CH_2N), 49.4 (1C, CH_2N), 55.8 (2C, CH_3O), 114.0 (4C, CH(Ph)), 114.8 (4C, CH(Ph)), 142.6 (1C, C1(Ph)), 142.7 (1C, C1(Ph)), 152.0 (2C, C4(Ph)); HRMS (MALDI-TOF) calcd. for $C_{21}H_{32}N_3O_2$ 358.2495, found 358.2481 $[M+H]^+$.

***N*^{1},*N*$^{1'}$-(Propane-1,3-diyl)bis(*N*^{3}-phenylpropane-1,3-diamine)** (**54**) was obtained from tetraamine **7** (1 mmol, 204 μL), iodobenzene (2.5 mmol, 280 μL) in the presence of CuI (19 mg) and L-proline (23 mg) in 2 mL EtCN. Eluent CH_2Cl_2/MeOH/NH_3(aq) 100:25:5. Yield 124 mg (37%), yellow viscous oil. ^{1}H NMR ($CDCl_3$) δ 1.83 (quintet, 4H, ^3J = 6.6 Hz, CCH_2C), 1.88 (quintet, 2H, ^3J = 5.9 Hz, CCH_2C), 2.79 (t, 4H, ^3J = 6.8 Hz, CH_2N), 2.88 (t, 4H, ^3J = 5.9 Hz, CH_2N), 3.13 (t, 4H, ^3J = 6.3 Hz, CH_2NPh), 4.63 (br.s, 2H, NHPh), 6.62 (d, 4H, $^3J_{obs}$ = 7.8 Hz, H2, H2'(Ph)), 6.67 (t, 2H, ^3J = 7.3 Hz, H4(Ph)), 7.41 (dd, 4H, $^3J_{obs}$ = 7.8 Hz, ^3J = 7.3 Hz, H3, H3'(Ph)), two NH protons of dialkylamino groups were not unambiguously assigned; ^{13}C NMR ($CDCl_3$) δ 25.8 (1C, C**CH_2**C), 27.9 (2C, C**CH_2**C), 41.9 (2C, CH_2NPh), 47.3 (2C, CH_2N), 49.1 (2C, CH_2N), 112.8 (4C, C2, C2'(Ph)), 117.3 (2C, C4(Ph)), 129.2 (4C, C3, C3'(Ph)), 148.2 (2C, C1(Ph)). MS (MALDI-TOF) calcd. for $C_{21}H_{33}N_4$ 341.2705, found 341.2663 $[M+H]^+$. By the treatment with 5M HCl solution in aqueous dioxane-methanol solution corresponding tetrahydrochloride was obtained as pale-beige crystalline powder, m.p. >240°C (decomp.). Calcd. for $C_{21}H_{36}Cl_4N_4$ (%): C 51.86, H 7.46, N 11.52; found C 52.39, H 7.84, N 10.99.

***N*^{1},*N*$^{1'}$-(Propane-1,3-diyl)bis(*N*^{3}-(biphenyl-4-yl)propane-1,3-diamine)** (**55**) was obtained from tetraamine **7** (1 mmol, 150 μL), 4-iodobiphenyl (2.5 mmol, 700 mg) in the presence of CuI (19 mg) and L-proline (23 mg) in 2 mL EtCN. Eluent CH_2Cl_2/MeOH/NH_3(aq) 100:20:3. Yield 119 mg (32%), pale-yellow crystalline powder, m.p. 108-110°C. ^{1}H NMR ($CDCl_3$) δ 1.51 (br.s, 2H, NH), 1.71 (quintet, 2H, ^3J = 6.8 Hz, C**CH_2**C), 1.82 (quintet, 4H, ^3J = 6.5 Hz, CCH_2C), 2.71 (t, 4H, ^3J = 6.9 Hz, **CH_2**$NHCH_2$), 2.77 (t, 4H, ^3J = 6.6 Hz, CH_2NH**CH_2**), 3.23 (t, 4H, ^3J = 6.6 Hz, CH_2NPh), 4.38 (br.s, 2H, NHPh), 6.65-6.68 (m, 4H, H(Ar)), 7.25 (tt, 2H, ^3J = 7.3 Hz, ^4J = 1.0 Hz, H(Ar)), 7.36-7.41 (m, 4H, H(Ar)), 7.42-7.46 (m, 4H, H(Ar)), 7.52-7.56 (m, 4H, H(Ar)); ^{13}C NMR ($CDCl_3$) δ 29.4 (2C, C**CH_2**C), 30.3 (1C, C**CH_2**C), 42.9 (2C, CH_2NPh), 48.4 (2C, **CH_2**$NHCH_2$), 48.5 (2C, CH_2NH**CH_2**), 112.9 (4C, CH(Ar)), 125.9 (2C, CH(Ar)), 126.2 (4C,

CH(Ar)), 127.8 (4C, CH(Ar)), 128.6 (4C, CH(Ar)), 129.9 (2C, C(Ar)), 141.3 (2C, C(Ar)), 148.0 (2C, NC(Ar)); HRMS (MALDI-TOF) calcd. for $C_{33}H_{41}N_4$ 493.3331, found 493.3359 $[M+H]^+$.

***N*^{1},*N*$^{1'}$-(propane-1,3-diyl)bis(*N*^{3}-(4-fluorophenyl)propane-1,3-diamine)** (**56**) was obtained from tetraamine **7** (0.74 mmol, 204 μL), 4-fluoroiodobenzene (2.5 mmol, 288 μL) in the presence of CuI (19 mg), 2-(isobutyryl)cyclohexanone (17 μL) and triphenylphosphine (26 mg) in 2 mL DMF. Eluent CH_2Cl_2/MeOH/NH_3(aq) 100:25:5. Yield 139 mg (37%), pale-yellow viscous oil. ^{1}H NMR ($CDCl_3$) δ 1.70 (quintet, 2H, ^3J = 6.8 Hz, C**CH₂**C), 1.77 (quintet, 4H, ^3J = 6.6 Hz, C**CH₂**C), 2.70 (t, 4H, ^3J = 6.7 Hz, **CH₂**NHCH₂), 2.73 (t, 4H, ^3J = 6.6 Hz, CH₂NH**CH₂**), 3.11 (t, 4H, ^3J = 6.6 Hz, CH_2NPh), 6.48-6.53 (m, 4H, $^4J_{HF}$ = 4.4 Hz, H2, H2'(Ph)), 6.82-6.88 (m, 4H, $^3J_{HF}$ = 8.7 Hz, H3, H3'(Ph)), NH protons were not unambiguously assigned; ^{13}C NMR ($CDCl_3$) δ 29.2 (2C, C**CH₂**C), 29.6 (1C, C**CH₂**C), 43.5 (2C, CH_2NPh), 48.3 (2C, **CH₂**NHCH₂), 48.6 (2C, CH₂NH**CH₂**), 113.5 (d, 4C, $^3J_{CF}$ = 6.8 Hz, C2, C2'(Ph)), 115.6 (d, 4C, $^2J_{CF}$ = 21.9 Hz, C3, C3'(Ph)), 144.9 (2C, C1(Ph)), 155.6 (d, 2C, $^1J_{CF}$ = 234.4 Hz, C4(Ph)); HRMS (MALDI-TOF) calcd. for $C_{21}H_{31}F_2N_4$ 377.2517, found 377.2476 $[M+H]^+$.

***N*^{1}-(3-aminopropyl)-*N*^{3}-(3-(4-fluorophenylamino)propyl)propane-1,3-diamine** (**57**) was obtained as the main product in the synthesis of compound **56** in the presence of CuI (19 mg) and L-proline (23 mg). Eluent CH_2Cl_2/MeOH/NH_3(aq) 10:4:1. Yield 87 mg (31%), yellow viscous oil. ^{1}H NMR ($CDCl_3$) δ 1.59-1.72 (m, 4H, C**CH₂**C), 1.77 (quintet, 2H, ^3J = 6.4 Hz, CH_2NPh), 2.64-2.80 (m, 10H, **CH₂**NH**CH₂**), 3.11 (t, 2H, ^3J = 6.5 Hz, CH_2NPh), 6.50 (dd, 2H, $^3J_{obs}$ = 8.6 Hz, $^4J_{HF}$ = 4.2 Hz, H2, H2'(Ph)), 6.84 (dd, 2H, $^3J_{obs}$ = 8.6 Hz, $^4J_{HF}$ = 8.6 Hz, H3, H3'(Ph)), NH and NH_2 were not unambiguously assigned; ^{13}C NMR ($CDCl_3$) δ 29.1 (2C, C**CH₂**C), 32.9 (1C, **CH₂**CNH₂), 40.3 (1C, CH_2NH_2), 43.5 (1C, CH_2NPh), 47.8 (1C, **CH₂**NHCH₂), 48.3 (3C, **CH₂**NH**CH₂**), 113.4 (d, 2C, $^3J_{CF}$ = 7.6 Hz, C2, C2'(Ph)), 115.5 (d, 2C, $^2J_{CF}$ = 22.8 Hz, C3, C3'(Ph)), 144.9 (1C, C1(Ph)); 155.6 (d, 1C, $^1J_{CF}$ = 234.4 Hz, C4(Ph)); HRMS (MALDI-TOF) calcd. for $C_{15}H_{28}FN_4$ 283.2298, found 283.2270 $[M+H]^+$.

***N*^{1},*N*$^{1'}$-(Propane-1,3-diyl)bis(*N*^{3}-(4-(trifluoromethyl)phenyl)propane-1,3-diamine)** (**58**) was obtained from tetraamine **7** (1 mmol, 204 μL), 4-iodo(trifluoromethyl)benzene (2.5 mmol, 367 μL) in the presence of CuI (19 mg) and L-proline (23 mg) in 2 mL EtCN. Eluent CH_2Cl_2/MeOH/NH_3(aq) 100:20:3. Yield 180 mg (38%), brown viscous oil. ^{1}H NMR ($CDCl_3$) δ 1.82 (quintet, 6H, ^3J = 6.5 Hz, C**CH₂**C), 2.80 (t, 4H, ^3J = 6.7 Hz, CH_2N), 2.84 (t, 4H, ^3J = 6.3 Hz, CH_2N), 3.18 (t, 4H, ^3J = 6.3 Hz, CH_2NPh), 4.43 (br.s, 2H NH); 4.96 (br.s, 2H, NHPh), 6.59

(d, 4H, $^{3}J_{obs}$ = 8.6 Hz, H2, H2'(Ph)), 7.35 (d, 4H, $^{3}J_{obs}$ = 8.6 Hz, H3, H3'(Ph)); ^{13}C NMR ($CDCl_3$) δ 26.9 (1C, C**C**H_2C), 27.9 (2C, C**C**H_2C), 41.7 (2C, CH_2NPh), 47.4 (2C, CH_2N), 49.0 (2C, CH_2N), 111.7 (4C, C2, C2'(Ph)), 118.4 (q, 2C, $^{2}J_{CF}$ = 32.0 Hz, C4(Ph)), 125.0 (q, 2C, $^{1}J_{CF}$ = 270.3 Hz, CF_3), 126.5 (4C, C3, C3'(Ph)), 150.8 (2C, C1(Ph)); HRMS (MALDI-TOF) calcd. for $C_{23}H_{31}F_6N_4$ 477.2453, found 477.2443 $[M+H]^+$. By the treatment with 5M HCl solution in aqueous dioxane-methanol solution corresponding trihydrochloride as solvate with methanol was obtained as beige crystalline powder, m.p. 180°C (decomp.). Calcd. for $C_{23}H_{33}Cl_3F_6N_4{*}CH_3OH$ (%): C 46.65, H 6.04, N 9.07; found C 46.27, H 5.97, N 8.49.

N^1,$N^{1'}$-(Propane-1,3-diyl)bis(N^3-(4-methoxyphenyl)propane-1,3-diamine) (**59**) was obtained from tetraamine **7** (0.5 mmol, 102 μL), 4-iodoanisole (5 mmol, 1170 mg) in the presence of CuI (19 mg) and 2-(isobutyryl)cyclohexanone (33 μL) in 3 mL DMF. Eluent CH_2Cl_2/MeOH/NH_3(aq) 100:25:5. Yield 33 mg (17%), brown viscous oil. ^{1}H NMR ($CDCl_3$) δ 1.76-1.85 (m, 6H, C**CH_2**C), 2.77 (t, 4H, ^{3}J = 6.7 Hz, CH_2N), 2.82 (t, 4H, ^{3}J = 5.8 Hz, CH_2N), 3.00 (br.s, 2H, NH), 3.08 (t, 4H, ^{3}J = 6.4 Hz, CH_2NPh), 3.70 (s, 6H, CH_3O) 4.55 (br.s, 2H, NHPh), 6.59 (d, 4H, $^{3}J_{obs}$ = 8.8 Hz, H2, H2'(Ph)), 6.75 (d, 4H, $^{3}J_{obs}$ = 8.8 Hz, H3, H3'(Ph)); ^{13}C NMR ($CDCl_3$) δ 28.4 (2C, C**CH_2**C), 31.4 (1C, C**CH_2**C), 43.2 (2C, CH_2NPh), 47.7 (2C, CH_2N), 48.9 (2C, CH_2N), 55.8 (2C, CH_3O), 114.2 (4C, CH(Ph)), 114.9 (4C, CH(Ph)), 142.5 (2C, C1(Ph)), 152.1 (2C, C4(Ph)); HRMS (MALDI-TOF) calcd. for $C_{23}H_{37}N_4O_2$ 401.2917, found 401.2944 $[M+H]^+$.

N^1,$N^{1'}$-(Butane-1,4-diyl)bis(N^3-phenylpropane-1,3-diamine) (**60**) was obtained from tetraamine **8** (1 mmol, 202 mg), iodobenzene (2.5 mmol, 280 μL) in the presence of CuI (19 mg) and 2-(isobutyryl)cyclohexanone (33 μL) in 2 mL DMF. Eluent CH_2Cl_2/MeOH/NH_3(aq) 10:4:1. Yield 245 mg (69%), pale-yellow viscous oil. ^{1}H NMR ($CDCl_3$) δ 1.60 (br.s, 4H, CCH_2CH_2C), 1.81 (quintet, 4H, ^{3}J = 6.3 Hz, CCH_2C), 2.64 (br.s, 4H, CH_2N), 2.75 (t, 4H, ^{3}J = 6.6 Hz, CH_2N), 3.15 (t, 4H, ^{3}J = 6.3 Hz, CH_2NPh), 3.76 (br.s, 4H, NH, NHPh), 6.60 (d, 4H, $^{3}J_{obs}$ = 7.8 Hz, H2, H2'(Ph)), 6.67 (t, 2H, ^{3}J = 7.2 Hz, H4(Ph)), 7.14 (t, 4H, $^{3}J_{obs}$ = 7.6 Hz, H3, H3'(Ph)); ^{13}C NMR ($CDCl_3$) δ 27.3 (2C, C**CH_2CH_2**C), 28.5 (2C, C**CH_2**C), 42.2 (2C, CH_2NPh), 47.4 (2C, CH_2N), 49.1 (2C, CH_2N), 112.7 (4C, C2, C2'(Ph)), 117.1 (2C, C4(Ph)), 129.1 (4C, C3, C3'(Ph)), 148.3 (2C, C1(Ph)); HRMS (MALDI-TOF) calcd. for $C_{22}H_{35}N_4$ 355.2862, found 355.2873 $[M+H]^+$. By the treatment with 5M HCl solution in aqueous dioxane-methanol solution corresponding tetrahydrochloride was obtained as pale-beige crystalline powder, t. decomp. 230°C. Calcd. for $C_{22}H_{38}Cl_4N_4$ (%): C 52.81, H 7.65, N 11.20; found 53.04, H 7.59, N 11.51.

***N*^{1},*N*$^{1'}$-(Butane-1,4-diyl)bis(*N*^{3}-(biphenyl-4-yl)propane-1,3-diamine)** (**61**) was obtained from tetraamine **8** (1 mmol, 202 mg), 4-iodobiphenyl (2.5 mmol, 700 mg) in the presence of CuI (19 mg) and L-proline (23 mg) in 2 mL EtCN. Eluent CH_2Cl_2/MeOH/NH_3(aq) 100:20:2 – 100:20:3. Yield 183 mg (36%), yellow viscous oil. ^{1}H NMR ($CDCl_3$) δ 1.53-1.59 (m, 4H, CCH_2CH_2C), 1.81 (quintet, 4H, ^3J = 6.5 Hz, CCH_2C), 2.61-2.67 (m, 4H, CH_2N), 2.76 (t, 4H, ^3J = 6.5 Hz, CH_2N), 3.23 (t, 4H, ^3J = 6.5 Hz, CH_2NPh), 4.41 (br.s, 2H, NHPh), 6.67 (d, 4H, $^3J_{obs}$ = 8.5 Hz, H(Ar)), 7.25 (t, 2H, ^3J = 7.3 Hz, H(Ar)), 7.39 (t, 4H, $^3J_{obs}$ = 7.6 Hz, H(Ar)), 7.44 (d, 4H, $^3J_{obs}$ = 8.6 Hz, H(Ar)), 7.54 (d, 4H, $^3J_{obs}$ = 7.5 Hz, H(Ar)), two NH protons of dialkylamino groups were not unambiguously assigned; ^{13}C NMR ($CDCl_3$) δ 27.9 (2C, CCH_2CH_2C), 29.4 (2C, CCH_2C), 42.9 (2C, CH_2NPh), 48.3 (2C, CH_2N), 49.8 (2C, CH_2N), 112.9 (4C, CH(Ar)), 125.9 (2C, CH(Ar)), 126.2 (4C, CH(Ar)), 127.9 (4C, CH(Ar)), 128.6 (4C, CH(Ar)), 129.9 (2C, C(Ar)), 141.3 (2C, C(Ar)), 148.0 (2C, NC(Ar)); HRMS (MALDI-TOF) calcd. for $C_{34}H_{43}N_4$ 507.3488, found 507.3445 $[M+H]^+$. By the treatment with 5 M HCl solution in aqueous dioxane-methanol solution corresponding tetrahydrochloride was obtained as yellow crystalline powder, m.p. >240°C. Calcd. for $C_{34}H_{46}Cl_4N_4$ (%): C 62.58, H 7.11, N 8.59; found C 62.35, H 7.09, N 8.25.

***N*^{1},*N*$^{1'}$-(Butane-1,4-diyl)bis(*N*^{3}-(4-fluorophenyl)propane-1,3-diamine)** (**62**) was obtained from tetraamine **8** (1 mmol, 202 mg), 4-fluoroiodobenzene (2.5 mmol, 288 μL) in the presence of CuI (19 mg) and 2-(isobutyryl)cyclohexanone (33 μL) in 2 mL DMF. Eluent CH_2Cl_2/MeOH/NH_3(aq) 10:4:1. Yield 175 mg (45%), pale-yellow viscous oil. ^{1}H NMR ($CDCl_3$) δ 1.56 (br.s, 4H, CCH_2CH_2C), 1.79 (quintet, 4H, ^3J = 6.4 Hz, CCH_2C), 2.63 (br.s, 4H, CH_2N), 2.74 (t, 4H, ^3J = 6.6 Hz, CH_2N), 3.11 (t, 4H, ^3J = 6.2 Hz, CH_2NPh), 3.31 (br.s, 4H, NH, NHPh), 6.51 (dd, 4H, $^3J_{obs}$ = 8.7 Hz, $^4J_{HF}$ = 4.3 Hz, H2, H2'(Ph)), 6.84 (dd, 4H, $^3J_{obs}$ = 8.7 Hz, $^3J_{HF}$ = 8.7 Hz, H3, H3'(Ph)); ^{13}C NMR ($CDCl_3$) δ 27.4 (2C, C**C**H_2**C**H_2C), 28.8 (2C, C**C**H_2C), 43.2 (2C, CH_2NPh), 47.8 (2C, CH_2N), 49.3 (2C, CH_2N), 113.4 (d, 4C, $^3J_{CF}$ = 6.7 Hz, C2, C2'(Ph)), 115.5 (d, 4C, $^2J_{CF}$ = 21.9 Hz, C3, C3'(Ph)), 144.8 (2C, C1(Ph)), 155.7 (d, 2C, $^1J_{CF}$ = 234.4 Hz, C4(Ph)); HRMS (MALDI-TOF) calcd. for $C_{22}H_{33}F_2N_4$ 391.2673, found 391.2696 $[M+H]^+$.

***N*^{1},*N*$^{1'}$-(Butane-1,4-diyl)bis(*N*^{3}-(4-(trifluoromethyl)phenyl)propane-1,3-diamine)** (**63**) was obtained from tetraamine **8** (1 mmol, 202 mg), 4-iodo(trifluoromethyl)benzene (2.5 mmol, 367 μl) in the presence of CuI (19 mg) and 2-(isobutyryl)cyclohexanone (33 μL) in 2 mL DMF. Eluent CH_2Cl_2/MeOH/NH_3(aq) 10:4:1. Yield 202 mg (41%), pale-yellow viscous oil. ^{1}H NMR ($CDCl_3$) δ 1.53-1.59 (m, 4H, CCH_2CH_2C), 1.79 (quintet, 4H, ^3J = 6.1 Hz, CCH_2C), 2.59-2.65 (m, 4H, CH_2NHCH_2), 2.75 (t, 4H, ^3J = 6.2 Hz, CH_2NHCH_2), 3.20 (t, 4H, ^3J = 6.3 Hz, CH_2NPh), 5.02 (br.s, 2H, NH), 6.56 (d, 4H, $^3J_{obs}$ = 8.3 Hz, H2, H2'(Ph)), 7.35 (d, 4H, $^3J_{obs}$ = 8.3 Hz, H3,

H3'(Ph)), two NH protons of dialkylamino groups were not unambiguously assigned; ^{13}C NMR ($CDCl_3$) δ 27.8 (2C, C**C**H_2**C**H_2C), 28.7 (2C, C**C**H_2C), 42.6 (2C, CH_2NPh), 48.2 (2C, **C**H_2NHCH_2), 49.6 (2C, CH_2NH**C**H_2), 111.6 (4C, C2, C2'(Ph)), 118.2 (q, 2C, $^2J_{CF}$ = 32.3 Hz, C4(Ph)), 125.0 (q, 2C, $^1J_{CF}$ = 270.2 Hz, CF_3), 126.5 (4C, C3, C3'(Ph)), 151.0 (2C, C1(Ph)); HRMS (MALDI-TOF) calcd. for $C_{24}H_{33}F_6N_4$ 491.2619, found 491.2588 $[M+H]^+$. By the treatment with 5M HCl solution in aqueous dioxane-methanol solution corresponding trihydrochloride as a solvate with methanol was obtained as beige crystalline powder, m.p. 200°C (decomp.). Calcd. for $C_{24}H_{35}Cl_3F_6N_4{*}CH_3OH$ (%): C 47.51, H 6.22, N 8.87; found C 48.16, H 6.59, N 8.80.

***N*^{1},*N*$^{1'}$-(Butane-1,4-diyl)bis(*N*^{3}-(4-methoxyphenyl)propane-1,3-diamine)** (**64**) was obtained from tetraamine **8** (1 mmol, 202 mg), 4-iodoanisole (2.5 mmol, 585 mg) in the presence of CuI (38 mg) and 2-(isobutyryl)cyclohexanone (67 μL) in 2 mL DMF. Eluent CH_2Cl_2/MeOH/NH_3(aq) 100:25:1 - 10:4:1. Yield 95mg (23%), yellow viscous oil. ^{1}H NMR ($CDCl_3$) δ 1.58-1.63 (m, 4H, CCH_2CH_2C), 1.80 (quintet, 4H, ^3J = 6.6 Hz, CCH_2C), 2.62-2.67 (m, 4H, CH_2N); 2.75 (t, 4H, ^3J = 6.8 Hz, CH_2N), 3.11 (t, 4H, ^3J = 6.4 Hz, CH_2NPh), 3.70 (s, 6H, OCH_3), 6.57 (d, 4H, $^3J_{obs}$ = 8.7 Hz, H2, H2'(Ph)), 6.74 (d, 4H, $^3J_{obs}$ = 8.7 Hz, H3, H3'(Ph)), NH protons were not unambiguously assigned; ^{13}C NMR ($CDCl_3$) δ 27.5 (2C, C**C**H_2**C**H_2C), 28.7 (2C, C**C**H_2C), 43.4 (2C, CH_2NPh), 47.6 (2C, CH_2N), 49.1 (2C, CH_2N), 55.8 (2C, CH_3O), 114.0 (4C, CH(Ph)), 114.8 (4C, CH(Ph)), 142.8 (2C, C1(Ph)), 152.0 (2C, C4(Ph)); HRMS (MALDI-TOF) calcd. for $C_{24}H_{39}N_4O_2$ 415.3073, found 415.3052 $[M+H]^+$.

References

1. Zhang, C.; Zhan, Z.; Lei, M.; Hu, L. *Tetrahedron.* **2014**, 70, 8817-8821.
2. Liu, S.; Rebros, M.; Stephens, G.; Marr, A.C. *Chem. Commun.* **2009**, 2308-2310.
3. Maruoka, K.; Banno, H.; Nonoshita, K.; Yamamoto, H. *Tetrahedron Lett.* **1989**, *30*, 1265-1266.
4. Ramirez, M. A.; Corona, M. V.; Blanco, M. M.; Perillo, I. A.; Porcal, W.; Salerno, A. *Tetrahedron Lett.* **2010**, *51*, 5000-5002.
5. Beletskaya, I. P.; Bessmertnykh, A. G.; Averin, A. D.; Denat, F.; Guilard, R. *Eur. J. Org. Chem.* **2005**, 261-280.
6. Vaultier, M.; Carboni, B.; Martinez-Fresneda, P. *Synth. Commun.* **1992**, *22*, 665-671.
7. Lang, F.; Zewge, D.; Houpis, I. N.; Volante, R. P. *Tetrahedron Lett.* **2001**, *42*, 3251-3254.
8. Blank, B.; Michlik, S.; Kempe, R. *Adv. Synth. Catal.* **2009**, *351*, 2903-2911.
